# Differential binding and co-binding pattern of FOXA1 and FOXA3 and their relation to H3K4me3 in HepG2 cells revealed by ChIP-seq

**DOI:** 10.1186/gb-2009-10-11-r129

**Published:** 2009-11-17

**Authors:** Mehdi Motallebipour, Adam Ameur, Madhu Sudhan Reddy Bysani, Kalicharan Patra, Ola Wallerman, Jonathan Mangion, Melissa A Barker, Kevin J McKernan, Jan Komorowski, Claes Wadelius

**Affiliations:** 1 Department of Genetics and Pathology, Uppsala University, Rudbeck Laboratory, Dag Hammarskjölds väg 20, Uppsala SE-75185, Sweden; 2 Linnaeus Centre for Bioinformatics, Uppsala University, Biomedical Center, Husargatan 3, Uppsala SE-75124, Sweden; 3 Applied Biosystems UK, 120 Birchwood Boulevard, Warrington WA3 7QH, Cheshire, UK; 4 Life Technologies, 850 Lincoln Centre Drive, Foster City, CA 94404, USA; 5 Life Technologies, 500 Cummings Center, Suite 2400, Beverly, MA 01915, USA; 6 Interdisciplinary Centre for Mathematical and Computer Modeling, Warsaw University, Krakowskie Przedmieœcie 26/28, Warszawa 00-927, Poland; 7 Current address: MRC Clinical Sciences Centre, Faculty of Medicine, Imperial College London, Hammersmith Hospital Campus, Du Cane Road, London W12 0NN, UK; 8 Current address: Department of Genetics and Pathology, Rudbeck Laboratory, Uppsala University, Dag Hammarskjölds väg 20, Uppsala SE-75185, Sweden; 9 Current address: Department of Development and Genetics, EBC, Uppsala University, Norbyvägen 18, Uppsala SE-75236, Sweden

## Abstract

**Background:**

The forkhead box/winged helix family members FOXA1, FOXA2, and FOXA3 are of high importance in development and specification of the hepatic linage and the continued expression of liver-specific genes.

**Results:**

Here, we present a genome-wide location analysis of FOXA1 and FOXA3 binding sites in HepG2 cells through chromatin immunoprecipitation with detection by sequencing (ChIP-seq) studies and compare these with our previous results on FOXA2. We found that these factors often bind close to each other in different combinations and consecutive immunoprecipitation of chromatin for one and then a second factor (ChIP-reChIP) shows that this occurs in the same cell and on the same DNA molecule, suggestive of molecular interactions. Using co-immunoprecipitation, we further show that FOXA2 interacts with both FOXA1 and FOXA3 *in vivo*, while FOXA1 and FOXA3 do not appear to interact. Additionally, we detected diverse patterns of trimethylation of lysine 4 on histone H3 (H3K4me3) at transcriptional start sites and directionality of this modification at FOXA binding sites. Using the sequence reads at polymorphic positions, we were able to predict allele specific binding for FOXA1, FOXA3, and H3K4me3. Finally, several SNPs associated with diseases and quantitative traits were located in the enriched regions.

**Conclusions:**

We find that ChIP-seq can be used not only to create gene regulatory maps but also to predict molecular interactions and to inform on the mechanisms for common quantitative variation.

## Abstract 2

FOXA1 and FOXA3 binding patterns in HepG2 cells, together with their possible molecular interactions with FOXA2 and each other, are revealed by ChIP-seq.

## Background

The forkhead box/winged helix (FOX) family of transcription factors (TFs) is conserved from yeast to mammals, and in humans consists of approximately 40 members [1-3]. A subfamily of these factors is the FOXA family with the members FOXA1 (formerly known as hepatocyte nuclear factor (HNF)3α), FOXA2 (HNF3β), and FOXA3 (HNF3γ), involved in development of the liver tissue and regulation of expression of the liver specific genes [4,5]. More specifically, FOXA1 and FOXA2 have been established as crucial for competence of the liver in the foregut endoderm during development [4]. This is suggested to be due to the ability of FOXAs to act as 'pioneering' factors opening the compacted chromatin [6]. FOXAs are also able to induce nucleosome positioning in a nucleosomal array, which has been demonstrated to occur in the enhancer region of the mouse serum albumin gene [7]. In an X-ray crystallographic study of FOXA3 bound to DNA, it was suggested that these factors bind as monomers and that the structure of FOXAs is similar to those of histones H1 and H5 [8]. The latter is proposed to be the explanation for the ability of FOXA to position nucleosomes and act as a pioneering factor [6].

FOXA1, -2, and -3 share great homology in the DNA binding domain. FOXA1 shares 95% and FOXA2 90% sequence identity with FOXA3 within the *forkhead *domain [8]. While FOXA1 and -2 are up to 39% identical outside of the *forkhead *domain, FOXA3 has much less similarity with these factors [2]. The FOXAs regulate genes involved in metabolism [2,5,9], for example, those encoding transthyretin, and apolipoproteins. Moreover, FOXA2 autoregulates its own expression and that of other TFs - for example, HNF4α, HNF1, and HNF6 - and has therefore been implicated as a master regulator of gene expression in the liver [9-11]. In the study by Duncan *et al*. [9], it was suggested that FOXA1 is a weaker transcription enhancer than FOXA2. It was further proposed that as FOXA1 and FOXA2 have the same recognition sequence on DNA, they compete for the binding site and FOXA1 may therefore exhibit an inhibitory effect.

There have been some chromatin immunoprecipitation (ChIP)-chip and ChIP with detection by sequencing (ChIP-seq) studies on members of the FOXA family published, specifically on FOXA1 (in MCF-7 and LNCaP cells) [12-14] and FOXA2 (mouse liver and a limited study in human liver) [15-17]. Although these studies have revealed interesting aspects of FOXAs as TFs, none have examined the interrelationship of the three members of this family. Additionally, several of these studies have only investigated the FOXA binding sites at the promoters of known genes and thus have not been truly genome-wide, despite the evidence that, for example, FOXA2 binds at sites other than the transcriptional start sites (TSSs) [16,18].

Modifications of the amino-terminal tails of the histones can change the accessibility of the chromatin for TFs and the transcriptional machinery and thereby regulate the expression of genes. Although combinations of these modifications are indicated as a prerequisite for activation or repression of the transcriptional activity [19], genome-wide studies of all the required modifications in every condition is not practical. Therefore, one modification can be chosen as representative for an active or inactive state of transcription. In this study, we have selected trimethylation of lysine 4 on histone H3 (H3K4me3), a commonly studied histone modification, as an indication of regions actively transcribed or poised to be transcribed [20].

The new generation of sequencers, generally known as high throughput sequencers, has made the detection of DNA resulting from ChIP for genome-wide studies easier and more cost-effective. In this study, we aimed to characterize the genome-wide binding sites of FOXA1 and FOXA3, for the first time, in the hepatocellular carcinoma cell line HepG2 through ChIP and sequencing on the SOLiD platform. Furthermore, we intended to examine their possible interactions with each other, with FOXA2, and their correlation with H3K4me3 and other TFs *in vivo*. We found that FOXA1 and FOXA3 have dissimilar distributions of binding sites in HepG2. Intriguingly, although there were sites of FOXA1 and FOXA3 co-binding together with FOXA2, FOXA1 and FOXA3 did not seem to interact *in vivo*. Furthermore, we discovered that trimethylation of lysine 4 at histone H3 reveals different patterns or 'signatures' depending on the promoter structure and transcriptional activity. Importantly, H3K4me3 was often found at a distance of about 200 bases from the sites of FOXA1-2-3 binding, frequently directed towards the nearest TSS. Finally, we demonstrate that ChIP-seq can be used for detecting allele-specific binding and candidate functional single nucleotide polymorphisms (SNPs).

## Results

### Overall data analysis

For the genome-wide analysis of FOXA1 and FOXA3 binding sites and regions of H3K4me3 in HepG2 cells, ChIPs and detection by a high throughput sequencer was performed. In order to get a detailed view of the regions of H3K4me3, we decided to treat the chromatin with micrococcal nuclease (MNase). MNase recognizes the naked DNA, which is not tightly wrapped around the nucleosomes, and digests it. This, in combination with the ChIP, will lead to nucleosome-sized DNA (147 bp) that can be sequenced by high throughput sequencers, resulting in a fine mapping of the H3K4me3 pattern in the genome. After alignment of the raw reads and calculation of overlap signals, we compared the results between the different libraries prepared for sequencing and detected a good correlation (Figure S1 in Additional data file 1). Thereafter, the aligned reads were merged, ordered on genomic positions, and extended by the average fragment size (Table S1 in Additional data file 1). We also sequenced a fraction of the input material, generated in the ChIPs, to use as a negative control for detection of regions where repeats may cause false positive overlap signals. Then we identified peaks with significant ChIP-enrichment by considering both the ChIP- and input signals.

**Figure 1:**
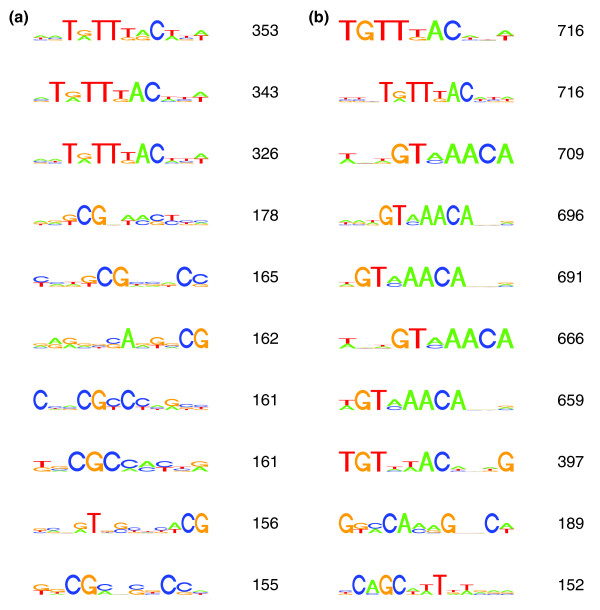
Results of *de novo *motif search. FOXA1 and FOXA3 data were analyzed using BCRANK as described in the Materials and methods. To the right of each motif is the assigned BCRANK score, which gives an indication of the quality of the motif. **(a) **Top ten predicted motifs for FOXA1. **(b) **Top ten predicted motifs for FOXA3.

We detected 8,175 peaks for FOXA1 and 4,598 peaks for FOXA3 in the human genome in the HepG2 cells (Table 1; files with information on peak positions for upload in the UCSC genome browser are available as Additional data files 2, 3, and 4). Out of these, only 465 (5.7%) and 562 (12.2%), respectively, were located within 1 kb of a TSS (Figure S2 in Additional data file 1), emphasizing the importance of true genome-wide studies for these factors. A majority of the putative binding sites were, as expected, located in intragenic and intergenic regions. Genes with a FOXA binding within 1 kb of their TSS demonstrated significantly higher expression than all genes (Figure S3 in Additional data file 1). A search with the *de novo *motif finding program BCRANK [21] resulted in different motifs with variations of TGTTTAC as the top three for FOXA1 and top eight for FOXA3 (Figure 1).

**Table 1:** Number of regions and overlaps with putative FOXA binding and H3K4me3

	FOXA1	FOXA2	FOXA3	H3K4me3
FOXA1	**8,175**	4,042 (49%)	3,065 (38%)	2,947 (36%)
FOXA2	4,025 (56%)	**7,153**	2,820 (39%)	2,796 (39%)
FOXA3	3,009 (65%)	2,775 (60%)	**4,598**	2,207 (48%)
H3K4me3	2,849 (7%)	2,560 (6%)	2,181 (5%)	**41,780**

As mentioned, members in the FOXA family regulate common pathways. This was supported by our Gene Ontology (GO) analysis, where some categories were recurrent for FOXA1 and FOXA3 (Figure S4A, B in Additional data file 1). Here, we consider a gene to be regulated by FOXA1 or FOXA3 when it contains a binding site within 1 kb of the TSS. Therefore, we analyzed the data for possible co-binding sites for these two factors. As presented in Table 1, more than 3,000 peaks were found in both data sets.

In a genome-wide study of FOXA1 binding sites in the human breast adenocarcinoma cell line (MCF-7), 12,904 regions have been found at a 1% false discovery rate [14]. Of these, 2,093 (16%) overlap with the putative binding sites found in HepG2 cells in our study. In a similar way, 2,178 (27%) of our regions were reciprocally found in the MCF-7-data. This indicates that around 2,000 FOXA1 binding sites are common between the HepG2 and MCF-7 cell lines, while 6,000 binding sites are unique to HepG2.

We found 41,780 H3K4me3 regions in the HepG2 genome (Table 1). This would approximately correspond to 160,000 nucleosomes with trimethylation of lysine 4 on histone H3. This number is calculated by multiplying the 41,780 regions by 764, which is the average peak length (Table S1 in Additional data file 1), and then dividing the product by 200, the assumed average distance in base-pairs between the start of two nucleosomes in these regions. Of the H3K4me3 regions, 42% are within 1 kb and an additional 15% within 5 kb of the TSS of a known gene, and 4.2% within 1 kb of a 3'-end (Figure S2 in Additional data file 1). Furthermore, 11% of these regions are intragenic, leaving 28% of the H3K4me3 not in the vicinity of a known gene.

### Distinct H3K4me3 at bidirectional and other promoter structures

**Figure 2:**
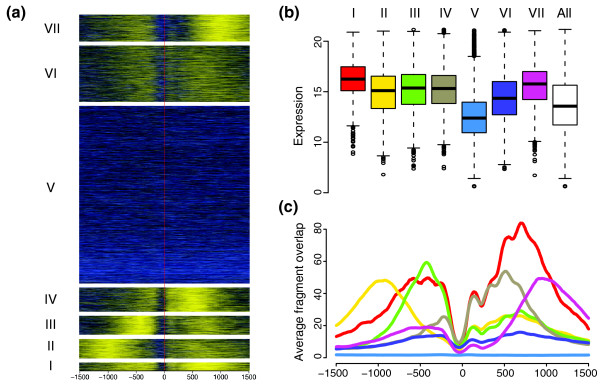
H3K4me3 signals around the transcriptions start sites of 23,849 genes. **(a) **Enrichment of H3K4me3 in a window surrounding the TSSs. The genes were grouped into seven clusters (I to VII) by their H3K4me3 patterns as described in the Materials and methods section. The enrichment scale is from high (yellow) to low (blue), and the red vertical line represents the TSS position. Negative x-coordinates are upstream of the TSS and positive are downstream. **(b) **Box plots indicating the distributions of expression levels in the seven clusters. The white box represents the expression for all genes. **(c) **Average H3K4me3 signal footprints for the seven clusters. The colors are as in (b).

Next, we aimed to discover patterns of H3K4me3 that could be indicative of different types of promoters. Therefore, we extracted the H3K4me3 signals around the TSSs of about 24,000 genes for which the expression measurements in HepG2 are available. We then performed k-means clustering of the H3K4me3 signals to partition the genes into seven clusters, each with its individual H3K4me3 signature (Figure 2a, c). Nearly all clusters seem to differ in the level of expression of the downstream genes from the other clusters (Figure 2b; Table S2 in Additional data file 1). Furthermore, comparison of the expression levels in each cluster to the expression of all 24,000 genes using a two-tailed *t*-test showed that all but cluster V have significantly higher expression than the average (*P *< 0.0001). Instead, cluster V has significantly lower expression than the average (*P *< 0.0001). Genes with the highest expression in HepG2 (cluster I with 596 genes; Table S3) tend to be more enriched for H3K4me3 than any other cluster. Opposed to this is cluster V (12,776 genes), which contains the lowest expressed genes with no or very low enrichment for H3K4me3. The common feature of the six clusters with high H3K4me3 levels is that a nucleosome with this modification was centered at approximately 125 bp downstream of the TSS. Furthermore, these six clusters also contained genes from different GO categories than those of cluster V, which had GO categories overrepresented for genes involved in development (Table S4 in Additional data file 1).

Considering clusters I, II, and III with high enrichments for H3K4me3 upstream of the TSS, we suspected the existence of bidirectional transcription in these regions. Therefore, the clusters were compared with the data for CAGE tags [22-24] in HepG2. CAGE (cap-analysis of gene expression) is a measurement of the expression of the TSSs of a gene. Consistent with our expectation, over 30% of the genes in each of these three clusters were in the vicinity of CAGE tags on the other strand compared to the TSS, that is, they were part of a bidirectional promoter (Table S3 in Additional data file 1). For cluster II, with a high and broad peak upstream of the TSS, this fraction exceeded 60%. Another significant finding was that 11% of the genes in cluster I, which had the highest expression, also had a FOXA3 binding site within 1 kb of their TSS (Table S3 in Additional data file 1).

Previous studies have suggested that bidirectional promoters occur in CpG-rich sequences [22,23]. Thus, we examined the frequency of different sequence elements at the TSSs of the genes in the seven clusters (Table S5 in Additional data file 1). Promoters for all clusters - except cluster V, which had the least number of bidirectional promoters - were highly enriched for CpG-rich sequences. As expected, cluster V contained a higher number of TATA- and CAAT-boxes.

Thus, by unsupervised clustering of enrichment signals around the TSSs, we detected different H3K4me3 signatures depending on the structure of the promoter, sequence elements present in the promoter, and the level of expression of the downstream gene. A similar type of analysis was also performed for H3K4me3 at the 3'-end of genes (Figure S5 in Additional data file 1). Some of the clusters with higher signals at the 3'-ends were associated with high expression of the gene, suggesting a reciprocal H3K4me3 signal at the beginning and the end of some genes. These clusters also have higher frequency of CAGE tags at the 3'-ends (Table S10 in Additional data file 1). For further comments, see the supplementary results in Additional data file 1.

### FOXA interactions detected by co-immunoprecipitation and ChIP-reChIP

**Figure 3:**
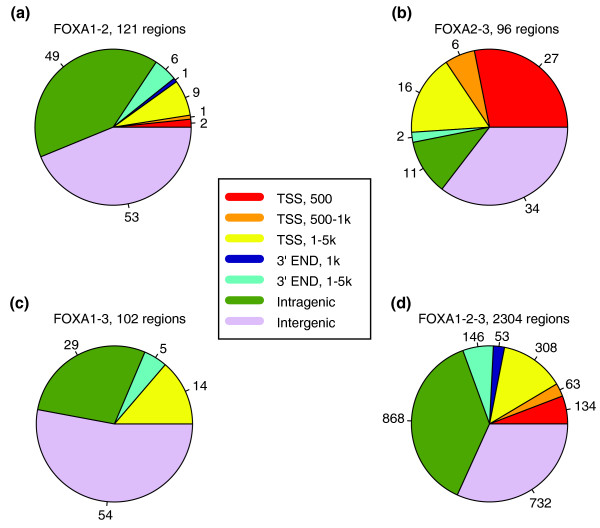
Genomic localization of common binding regions for FOXA1, FOXA2, and FOXA3. **(a) **FOXA1-2, **(b) **FOXA2-3, **(c) **FOXA1-3, and **(d) **FOXA1-2-3. Each region was mapped to all UCSC gene coordinates and sequentially matched to the categories 500 bp from TSS, 500 bp to 1 kb from TSS, 1 to 5 kb from TSS, 1 kb from 3'-end, 1 to 5 kb from 3'-end and intragenic. The intergenic group consists of those regions not matching any of the mentioned categories.

We have previously examined the genome-wide location of FOXA2 binding in HepG2 cells, where we found 7,253 binding sites for this factor [25]. Comparison of the FOXA2 data with that for FOXA1 and FOXA3 revealed 2,304 regions in common for all three factors. Here, a common binding is reported when the distance between the peak centers is less than 1 kb. Furthermore, when the genomic localization of different combinations of these factors was examined, we found around 100 regions of common binding for each pair (Figure 3). While 12 of 121 (10%) FOXA1-2 regions were within 5 kb of a TSS of a known gene (Figure 3a), 49 of 96 (51%) FOXA2-3 regions were within the same distance (Figure 3b). For FOXA1-3, 14 of 102 (14%) regions are within 5 kb, although there are no common binding sites for this pair within the first kilobase of a TSS (Figure 3c). The corresponding number for all three factors together is 22% (505 of 2,304; Figure 3d).

**Figure 4:**
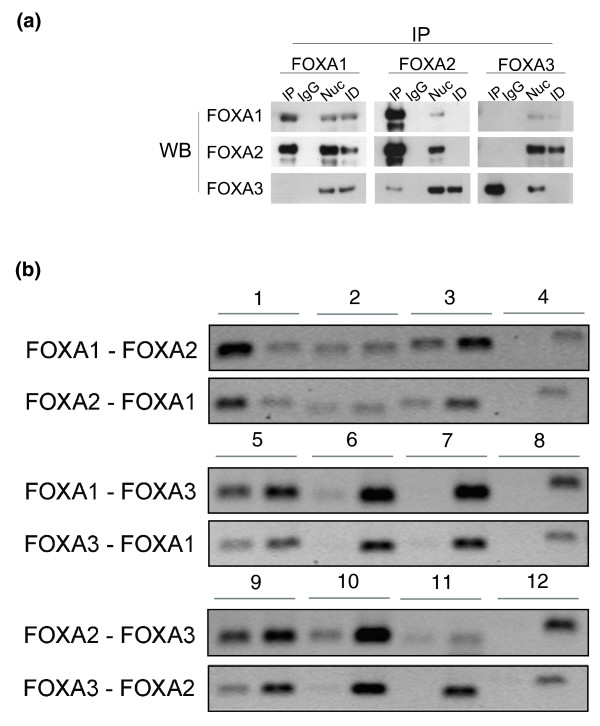
Co-immunoprecipitation and ChIP-reChIP of FOXAs reveals interaction and co-binding among FOXAs. **(a) **Immunoprecipitations were performed with indicated antibodies on nuclear extracts of HepG2 cells and the immunocomplexes were detected with FOXA1, FOXA2, and FOXA3 antibodies. IP, immunoprecipitation; IgG, the antibody was replaced by normal IgG; Nuc, total nuclear extract; ID, immunodepleted fraction obtained after IP. The blots are representative of two or three replicates. None of the proteins was overexpressed. **(b) **ChIP-reChIP of FOXA1, FOXA2, and FOXA3 tested by semiquantitative PCR. The order of antibodies used to immunoprecipitate the protein-DNA complex is indicated to the left. In each pair of bands, the left one is for the IP and the right for input. Pairs 1, 5, and 9: a primer amplifying a region with binding site for both proteins; pairs 2 and 6: regions with binding sites for FOXA1, but not FOXA2 or FOXA3, respectively; pairs 3 and 10: regions with binding sites for FOXA2, but not FOXA1 or FOXA3, respectively; pairs 7 and 11: regions with binding sites for FOXA3, but not FOXA1 or FOXA2, respectively; pairs 4, 8, and 12: a region with no FOXA binding. FOXA2-FOXA1, FOXA3-FOXA1, and FOXA3-FOXA2 were performed as independent experiments from the other three ChIP-reChIPs.

Based on these data, we assumed that FOXA1, FOXA2, and FOXA3 interact with each other *in vivo*. Therefore, we employed co-immunoprecipitation (Co-IP) to examine the existence of these complexes in HepG2. For this, we immunoprecipitated the three endogenous factors and immunoblotted with the same antibodies, testing all six possible combinations. We found that FOXA2 interacts with FOXA1 and the data suggest an interaction between FOXA2 and FOXA3 as well (Figure 4a). We could not detect any direct protein-protein interaction between FOXA1 and FOXA3.

The lack of evidence for a direct FOXA1 and FOXA3 interaction could be for technical reasons with regard to the Co-IP protocol. Therefore, to detect and verify possible co-bindings and to further understand whether these are due to binding of different FOXA molecules at the same site in different cells or due to co-binding in the same cell, we employed the ChIP-reChIP method in combination with semiquantitative PCR. With this method, crosslinked protein-DNA complexes are immunoprecipitated first with the antibody for one protein in the complex, followed by immunoprecipitation with the antibody for the second protein. We immunoprecipitated the chromatin from HepG2 cells with any of the three FOXA antibodies (FOXA1, FOXA2, and FOXA3) and reimmunoprecipitated the material with another of the three antibodies. The sequence of the pairs was then reversed in independent replicates in order to verify the results from the first round. The resulting DNA was then analyzed by PCR with primers amplifying a region containing enriched peaks for both factors in the complex. As a negative control, we used primers for regions containing a binding site for only one of the factors in the pair and primers for a region with no binding site for any of the factors. Theoretically, if two of the factors co-bind in a region, that sequence should be enriched in the ChIPed DNA, while sequences with a single binding should not be enriched as they are selected against by the serial immunoprecipitation. As demonstrated in Figure 4b, we could find that each of the factors FOXA1, FOXA2, and FOXA3 bind in close vicinity of any of the other two FOXAs on the same DNA molecule in the same cell.

With these results, we demonstrate regions of pair-wise binding for FOXA1, FOXA2, and FOXA3, where these factors co-bind in close proximity and, as indicated by the Co-IP data, some of these factors may even interact at the site of binding.

### Correlation of FOXA binding and H3K4me3

**Figure 5:**
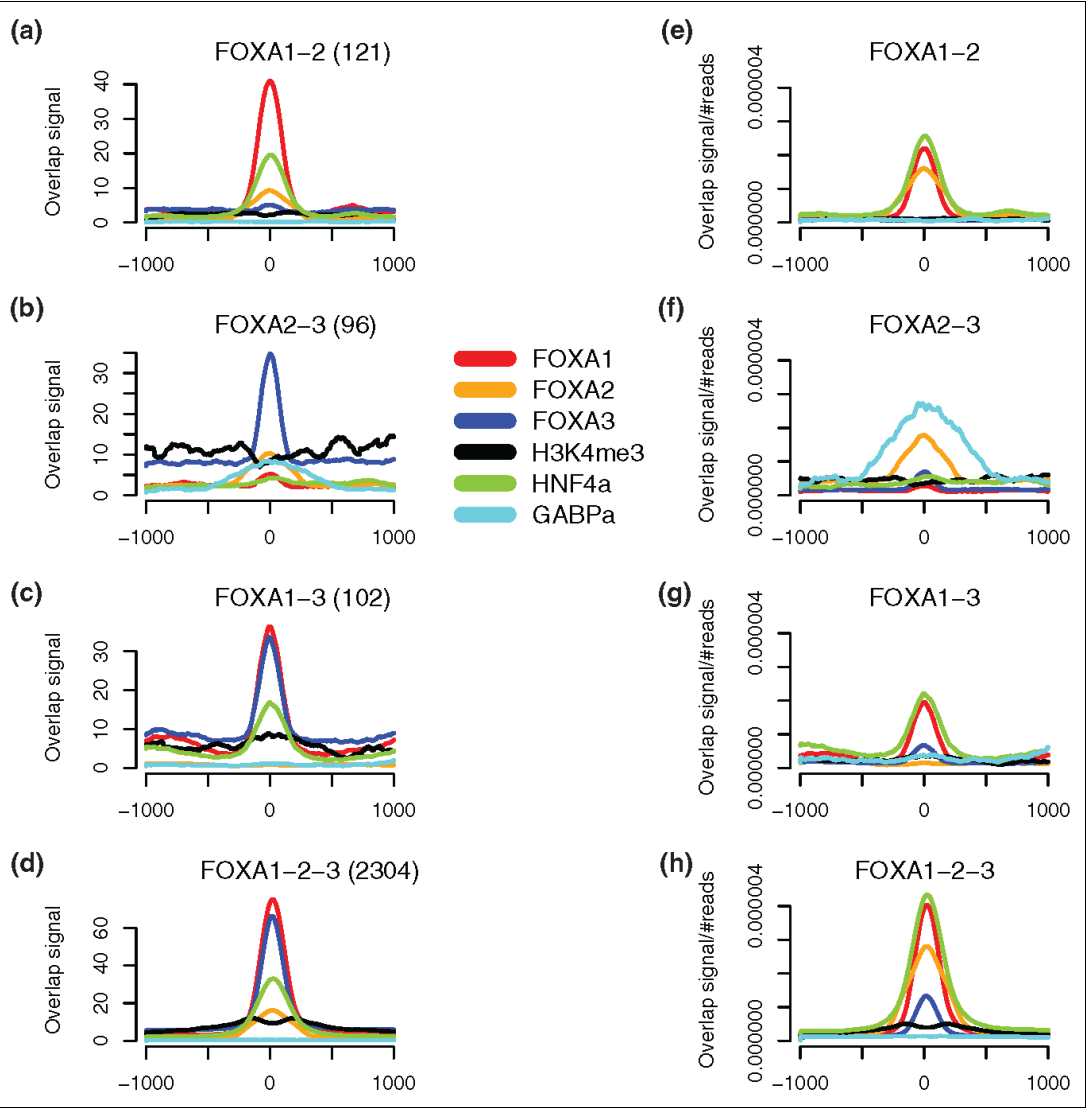
Enrichment signals in regions of pair-wise co-binding for FOXA1, FOXA2, and FOXA3. For each FOXA co-binding site, enrichment signals for FOXA1 (red), FOXA2 (orange), FOXA3 (blue), H3K4me3 (black), HNF4α (olive green), and GABP (turquoise) are plotted, centered on the putative FOXA binding site. **(a-d) **Graphs of the non-normalized data. **(e-h) **Graphs for each factor normalized to their number of aligned reads. Numbers in brackets for (a-d) are the number of sites with co-binding, as presented in Figure 3.

FOXA TFs are known to be involved in opening of compacted chromatin. Accordingly, we examined the H3K4me3 footprint pattern in the regions with FOXA1-2-3, FOXA1-2, FOXA2-3, and FOXA1-3 binding. Regions with FOXA1-2 and FOXA1-3 binding seem to have a lower enrichment for H3K4me3 than FOXA2-3 regions (Figure 5a-c, e-g). This was expected, as only 32% of FOXA1 binding sites had a region with H3K4me3 within 1 kb, compared to 44% for FOXA3 (Table 1). The more interesting finding is the pattern of histone trimethylation in regions with FOXA1-2-3 binding, where a double peak surrounds the peak of TF binding (Figure 5d, h).

**Figure 6:**
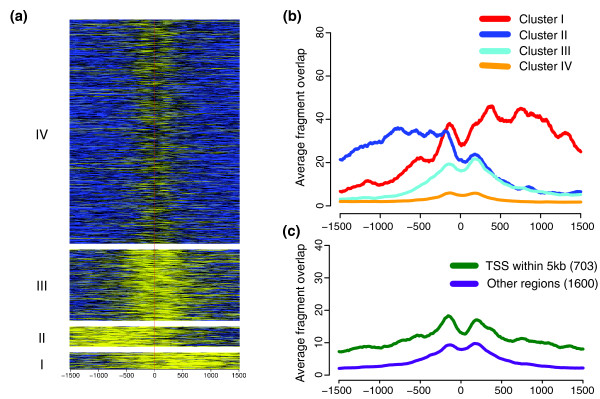
H3K4me3 signals around 2,303 FOXA1-2-3 regions. **(a) **Enrichment of H3K4me3 in a window surrounding the center of FOXA1-2-3 regions. The regions were grouped into four clusters (I to IV) by their H3K4me3 patterns. The enrichment scale is from high (yellow) to low (blue), and the red vertical line represents the FOXA1-2-3 centers. Negative x-coordinates are upstream of the centers and positive are downstream. **(b) **Average H3K4me3 signal footprints for the four clusters in (a). **(c) **Average H3K4me3 signal footprints for regions with a TSS within 5 kb independent of direction (green) and regions lacking a TSS within this distance (purple).

We looked further into this double peak by k-means clustering of the signal for H3K4me3 in four different clusters (Figure 6a, b). Two of these clusters, clusters I and II, revealed patterns that resembled those at the TSS (Figure 2c), with each of the curves on either side of the FOXA1-2-3 binding. Due to the observed pattern, we decided to look for TSSs within a 5 kb distance from the combined FOXA1-2-3 binding site. Of the 2,304 regions with triple binding, 505 contained a known TSS within this distance (Figure 3d), with a similar number of TSSs on the two strands (Table S6 in Additional data file 1). A majority of regions in clusters I and II were within 5 kb of a TSS and these clusters showed the highest levels of H3K4me3. The H3K4me3 peaks in these clusters are located at opposite sides of the FOXA1-2-3 binding, and this would suggest that the H3K4me3 signals are biased towards the direction of transcription (Figure 6b; Table S6 in Additional data file 1).

In the next step, we correlated these clusters with CAGE tags from HepG2 within the same distance as above. This comparison revealed a higher percentage of TSSs near the combined FOXA binding sites (Table S7 in Additional data file 1). When considering CAGE-tags within 1 kb, the difference in directionality for clusters I and II became more evident, with more CAGE tags in the plus direction for cluster I and in the minus direction for cluster II. Furthermore, by creating separate footprints of H3K4me3 around the FOXA1-2-3 regions with or without a TSS within 5 kb, we observed that both groups exhibit a double peak, each peak with its centre at a distance of approximately 200 bp from the binding site (Figure 6c). The H3K4me3 pattern around FOXA1-2-3 binding sites, as presented in our study, correlates well with the hypothesis that FOXAs position nucleosomes at their binding site [7], which is best supported by FOXA1-2-3 regions with a TSS within 5 kb (Figure 6c).

### Correlation of FOXA binding with other transcription factors

As mentioned previously, FOXAs are involved in auto- and feed-forward regulation of *FOXA *genes and other TF genes in the liver. Therefore, we examined the binding pattern at the *FOXA *genes and compared this with our data on upstream stimulatory factor (USF)1 and USF2 [26], and HNF4α and GABP (GA binding protein; NRF2) [25]. While *FOXA1 *and *FOXA2 *had binding sites for all three factors, *FOXA3 *does not seem to be regulated by any of the FOXAs (Figure S6 in Additional data file 1). Moreover, FOXA1 and FOXA3 both seem to co-bind with the other factors at a similar rate (Table 2). When we examined the co-binding of GABP with FOXA1-2, FOXA2-3, and FOXA1-3, we found that only the second complex had co-binding with it (Table 2 and Figure 5a-c, e-g). In our ChIP-seq study of GABP, we found that 85% of its putative binding sites were located at TSSs.

**Table 2:** Overlap between putative FOXA binding sites and the binding of other factors

	Number of regions	HNF4α*	GABP^†^	USF1^‡^	USF2
FOXA1	8,175	5,043 (62%)	47 (0.6%)	232 (2.8%)	288 (3.5%)
FOXA2	7,153	3,838 (54%)	158 (2.2%)	257 (3.6%)	274 (3.8%)
FOXA3	4,598	2,536 (55%)	147 (3.2%)	186 (4.0%)	227 (4.9%)
(FOXA1+FOXA2)-FOXA3	121	75 (62%)	0 (0%)	3 (2.5%)	3 (2.5%)
(FOXA2+FOXA3)-FOXA1	96	16 (17%)	22 (22.9%)	4 (4.2%)	4 (4.2%)
(FOXA1+FOXA3)-FOXA2	102	27 (26%)	0 (0%)	2 (2%)	3 (3%)
FOXA1+FOXA2+FOXA3	2,304	1,762 (76%)	31 (1.3%)	99 (4.3%)	140 (6.1%)

*Hepatocyte nuclear factor 4a. ^†^GA binding protein. ^‡^Upstream stimulatory factor.

Another interesting observation was that FOXA1-2 and FOXA1-3 regions were more related to HNF4α binding than FOXA2-3 (Table 2 and Figure 5a-c, e-g). In addition, FOXA1-2-3 binding is highly correlated with HNF4α binding in HepG2 cells (Table 2 and Figure 5d, h).

### Allele-specific DNA-protein interactions

Monoallelic expression of genes can be due to imprinting, allelic exclusion or sex chromosome dosage compensation. SNPs in combination with the ChIP-seq could prove to be a powerful method for detection of allele-specific binding that could lead to monoallelic or preferential expression from one allele in the studied genome. With a high enough number of sequence reads at a locus with a heterozygous SNP, one can detect whether the majority of reads are from one allele or the other. If TF binding or active histone marks are predominantly found on only one of the alleles, one can suspect preferential binding to that particular allele of the gene. Previously, we have interrogated the genome of HepG2 cells for SNPs by the Infinium assay and Human-1M array (Illumina) in 1,000,000 positions (data not shown). Among these, 220,000 were heterozygous SNPs (Additional data file 5), which we screened in the ChIP-seq data for allele-specific binding. After taking multiple testing into account as described in the Materials and methods section, we found three examples for FOXA1, two for FOXA3, and six for H3K4me3 (Table S8 in Additional data file 1).

**Figure 7:**
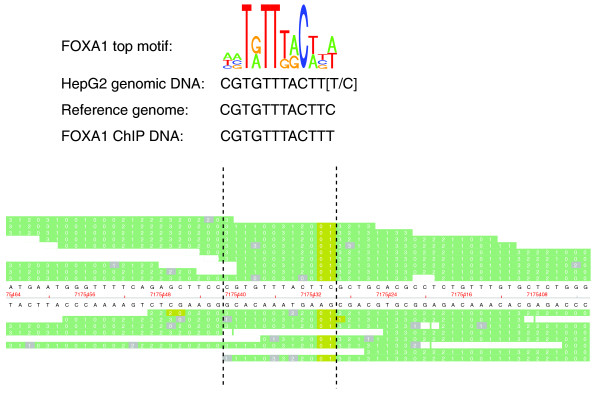
Preferential binding of FOXA1 at a heterozygous SNP. SNP rs7248104 is located in a FOXA1 binding sequence and FOXA1 is preferentially bound to one allele. At the top is the FOXA1 motif, predicted by the BCRANK method, followed by the sequence found in HepG2 with the alleles of the heterozygous SNP (T/C) in brackets. These are followed by the sequence in the reference genome and the sequence found in the FOXA1-reads. At the SNP position, the T-allele corresponds to the FOXA1 motif, which is found in all 15 FOXA1 reads, while the C-allele in the reference genome is not detected at all. The raw data for individual FOXA1 reads in the region are presented at the bottom, viewed in the SOLiD™ Alignment Browser tool. The positions marked in green correspond to bases (in the SOLiD™ two-base encoding) that align to the reference genome. In gray are the bases with a match error. The yellow bases correspond to positions with valid adjacent mismatches, indicating the locations of SNPs. The vertical hatched lines enclose the position of the motif.

A detailed view of the most significant SNP for FOXA1, rs7248104, located in an intronic region of the insulin receptor precursor gene (*INSR*), revealed some interesting results (Figure 7). rs7248104 is a heterozygous (C/T) SNP in HepG2 located in a DNA sequence that exactly matches the top motif found for FOXA1 (Figure 1a). The motif predicts binding of FOXA1 to the T-allele, but not the C-allele, which was reflected in the ChIP-seq data as all 15 reads that cover the SNP contain the T-allele (Figure 7). This could indicate rs7248104 as a functional SNP, due to its effect on FOXA1 binding to the DNA, although experimental data are required to confirm this.

### Combining ChIP-seq and SNP association data

Several genome-wide association studies have identified SNPs associated with various traits. Combining such data with our genome-wide DNA-protein interaction maps could offer a possibility to find functional SNPs. Here, we compared our data for FOXA1, FOXA3, and H3K4me3 with previously published genome-wide association studies for plasma levels of liver enzymes and metabolic traits, for example, lipid and fasting glucose levels [27-32]. We searched for these reported SNPs in all our positive regions and identified those that were associated with a specific trait and that were included in our significant peaks for TF binding or H3K4me3 (Table 3; Table S9 in Additional data file 1). Locating these SNPs in the regulatory regions is an important first step towards identification of functional SNPs and a possible hint on the effect of this nucleotide variation.

**Table 3:** Comparison of ChIP-seq data with genome-wide association studies for identification of functional SNPs

Chr.	Position	SNP	Nearest/affected gene	Factor	Trait	Study
1	109,619,113	rs12740374	*CELSR2, PSRC1, SORT1*	FOXA1	LDL*/Dyslipidemia	Kathiresan et al.
10	101,851,425	rs11597390	*CPN1*	FOXA1, FOXA3	Plasma levels of liver enzymes (ALT^†^)	Yuan et al.
1	109,620,053	rs646776	*CELSR2-PSRC1-SORT1*	H3K4me3	TC^‡^/LDL	Aulchenko et al., Sabatti et al.
2	43,918,594	rs6756629	*ABCG5*	H3K4me3	TC/LDL	Aulchenko et al.
11	47,226,831	rs2167079	*NR1H3*	H3K4me3	HDL^§^	Sabatti et al.
11	61,353,788	rs174570	*FADS2/3*	H3K4me3	TC/LDL	Aulchenko et al.
19	11,063,306	rs6511720	*LDLR*	H3K4me3	LDL/Dyslipidemia	Kathiresan et al.
19	50,087,106	rs157580	*TOMM40-APOE, APO *cluster	H3K4me3	TC/TG/LDL	Aulchenko et al., Sabatti et al.
19	50,087,459	rs2075650	*TOMM40-APOE*	H3K4me3	TC/LDL	Aulchenko et al.
22	23,320,213	rs4820599	*GGT1*	H3K4me3	Plasma levels of liver enzymes (GGT^¥^)	Yuan et al

*LDL, low density lipoproteins. ^†^ALT, alanine-aminotransferase. ^‡^TC, total cholestrol. ^§^HDL, high density lipoproteins. TG, triglycerides. ^¥^GGT, gamma-glutamyl transferase.

## Discussion

In this paper, we present the first true genome-wide location analysis of FOXA1 and FOXA3 binding sites in the human HepG2 cell line through ChIP-seq and their internal association. Our analysis demonstrates that among the FOXA family, FOXA1 is the more frequent binder with a majority of binding sites far from known genes, while FOXA3 binds least frequently and preferentially at sites near a known gene. Additionally, from Co-IP analyses we found that FOXA2 interacts with both other FOXAs, while FOXA1 and FOXA3 do not seem to interact. Through ChIP-reChIP experiments, we demonstrated pair-wise co-binding of the FOXA factors to the same sites of DNA in the same cell. These data were further substantiated by the differential binding pattern of these complexes and their interactions with other TFs located either at TSSs or at distant sites.

Out of the ten top-ranked motifs found for FOXA1 and FOXA3, only the few top motifs are canonical and the rest are either variations of the top-motif or other motifs. We have previously suggested that these non-canonical bindings might be due to interactions of FOXAs with other TFs and that these motifs might in fact be canonical motifs for the binding partners of FOXAs [18]. A recent report implies that different sequences at binding sites might affect the binding and regulatory activity of the interacting TF [33].

We did not find any evidence of protein-protein interaction between FOXA1 and FOXA3, but we cannot yet completely exclude a direct/indirect interaction. If there are any interactions between these two factors, they might be transient and rapid in the cells. These interactions might also be very weak and therefore easily lost during the treatments and washes, and hence not detected by Co-IP. Instances where FOXA1, FOXA2, and FOXA3 are found together could be due to two molecules of FOXA2 binding at the site and each recruiting one of the other two factors. Another possibility is the involvement of other factors, such as HNF4α, in engaging the different participants of the complex. Indeed, we demonstrate here that 76% of FOXA1-2-3 bindings coincide with HNF4α-binding. Previously, we have also detected that HNF4α co-immunoprecipitates with FOXA2 in HepG2 cells [25].

Based on the X-ray crystallographic structure of FOXA3, it was postulated that FOXAs bind DNA as monomers [8]. This is not in conflict with our results, as most of the binding sites for the FOXAs seem to contain only one motif for the FOXA factors. Regions with binding of two or all three factors could be through binding of one to the main motif and the second and/or third factor to other motifs. This is less likely as at the sites of interaction we mostly find a single peak for each of the factors, all in nearly the same position. We therefore hypothesize that each of the factors bind DNA as monomers at different positions and then may interact in diverse constellations through long-range interactions. Further studies are required to understand the nature of these interactions and the inter-relationship of the FOXA factors.

In this study, we have also mapped the histone modification H3K4me3 genome-wide in HepG2 cells. As in other studies, we find this modification mainly around the TSS of genes [34]. However, by clustering of data by k-means in these regions, we were able to discover several different patterns of H3K4me3, few of them highly correlated with the expression level of the gene and the surrounding promoter structure. Using the same approach, we could also detect five clusters at the 3'-end of genes. Again, here we found patterns that could be related to the structure of the DNA at these sites. Convergent or tail-to-tail genes, for example, exhibited other patterns than tail-to-head genes or genes without any other transcription units in the vicinity. Our results clearly demonstrate the importance of cautious interpretation of histone mark signals, as the average signal, usually displayed by footprints, is composed of a number of diverse patterns from subgroups of genes with different features.

FOXA TFs are demonstrated to act as pioneering factors, that is, they are able to bind to compacted chromatin and facilitate opening of the chromatin and recruitment of other TFs. We found here that binding of FOXA2-3 correlates more often with H3K4me3 than FOXA1-2 and that FOXA3 binding was associated with high expression and high enrichment for trimethylation of K4 on histone H3. Additionally, sites with triple binding of FOXAs had one or two peaks of H3K4me3 on either side of the binding site, with directionality towards a TSS. This can be interpreted as the binding of FOXAs upstream of a TSS positioning the nucleosomes in between, regardless of the distance, and recruiting the histone methyl-transferases that methylate the first nucleosome adjacent to the FOXA binding site. This mark may then be propagated to some extent between the binding site and the TSS.

Preferential binding of TFs and histone modifications to one allele of a SNP can easily be detected through ChIP-seq if there is a sufficient number of overlapping reads at heterozygous SNPs. Specifically, the preferred binding/modification can occur in regulatory elements with SNPs that are associated with common disorders. We found several examples of SNPs that were related to a preferential binding/modification in this study. Examination of these SNPs and their possible effect on the expression of nearby genes can offer mechanistic insights into inherited variation in gene expression and pathogenesis of common disorders.

## Conclusions

ChIP-seq is a powerful strategy that can be used for purposes other than to create gene regulatory maps. By combining data for several TFs, we predicted protein-protein interactions and sites of co-binding, which were validated in Co-IP and ChIP-reChIP experiments. By reading the sequence in ChIP-DNA at polymorphic sites, we find many instances of allele-specific DNA-protein interactions, which are important data to understand the mechanisms of allelic imbalance in gene expression. SNPs associated with diseases and common phenotypes are also frequently found in the enriched regions. This means that ChIP-seq is an additional high-throughput method to generate systematic data on the molecular basis of quantitative human variation.

## Materials and methods

### Chromatin immunoprecipitation - ChIP and ChIP-reChIP

The human hepatocellular carcinoma cell line HepG2 was cultured in RPMI-1640 with non-inactivated fetal bovine serum and penicillin-streptomycin at 37°C with 5% CO_2_. For the ChIP-assay, cells were washed and serum-free medium was added before incubation with formaldehyde in a final concentration of 0.37% for 10 minutes. Cells were then lysed and the nuclei were resuspended in RIPA-buffer (1× phosphate-buffered saline (PBS), 1% NP-40, 0.5% Na-deoxycholate, 0.1% SDS, 0.004% Na-azide) for sonication with a Bioruptor (Diagenode, Liège, Belgium) to obtain fragments with a size range between 150 and 300 bp. Sonicated chromatin was then pre-cleared and incubated with the antibody overnight. After a short incubation with Protein-G agarose (Roche, Mannheim, Germany), the chromatin-antibody-bead complex was washed four times with RIPA-buffer, once with 0.01 M Tris-HCl (pH 8), 0.25 M LiCl, 0.001 M EDTA, 1% NP-40 and 1% Na-deoxycholate, and once with TE-buffer. Then the chromatin was eluted with 1% SDS and 0.1 M NaHCO_3 _before RNase-treatment and reversal of the cross-linking at 65°C for 6 hours and Proteinase-K treatment overnight. The DNA was eluted with phenol-chloroform and precipitated with ethanol. Enrichment for each antibody was tested by semiquantitative PCR with primers for known binding sites.

ChIP-reChIP was performed as described in [35], except that after the first elution the material was diluted five times.

Antibodies used in this study were for FOXA1 (ab5089, Abcam, Cambridge, UK), FOXA2 (sc-6554, Santa Cruz Biotechnology, Santa Cruz, CA, USA), FOXA3 (sc-5361, Santa Cruz Biotechnology), and tri-methylation-histone H3 (K4) (05-745 (CL MC315100), Upstate, Temecula, CA, USA).

### Micrococcal nuclease-ChIP

Pelleted HepG2 cells were resuspended in ice-cold buffer A containing 320 mM sucrose, 15 mM HEPES pH 7.9, 60 mM KCl, 2 mM EDTA, 0.5 mM EGTA, 0.5% bovine serum albumin, 0.5 mM spermidine, 0.15 mM spermine, and 0.5 mM DTT. After a short incubation on ice, cells were homogenized with a Dounce homogenizer. The homogenate was then layered on an equal volume of buffer B, which was the same buffer as A except that it did not contain bovine serum albumin and the sucrose concentration was 30%. Collected nuclei were washed once with and resuspended in 0.34 mM sucrose, 15 mM HEPES pH 7.5, 60 mM KCl, 15 mM NaCl, 0.5 mM spermidine, 0.15 mM spermine, and 0.15 mM β-mercaptoethanol. After adjustment of CaCl_2 _concentration to 3 mM, the suspension was aliquoted to have 30 × 10^6 ^cells per milliliter. These were incubated at 37°C for 5 minutes before addition of 300 U of micrococcal nuclease to each aliquot, after which the incubation was continued for another 5 minutes. To stop the reaction 90 mM HEPES pH 7.9, 220 mM NaCl, 10 mM EDTA, 2% Triton X-100, 0.2% Na-deoxycholate, 0.2% SDS, 0.5 mM PMSF, and 2 μg/ml aprotinin was added. The solution was centrifuged and the supernatant was used to set up the ChIP-assay as described above.

### Library preparation and sequencing

All preparations of libraries for sequencing and the sequencing itself were according to the SOLiD™ System 2.0 Fragment Library Preparation: Lower Input DNA and User Guide standard protocols, with the following modifications.

For the FOXA1 library three ChIPs were performed, resulting in five immunoprecipitations, which were pooled. The majority of the fragments were in the size range of 150 to 300 bp after sonication during the ChIP. Therefore, to obtain a homogeneous fragment size and to avoid biased fragment amplification, we decided to prepare three fragment libraries. After ligation of adapters and purification, the DNA was separated by PAGE and three fragment size ranges selected: 150 to 200 bp, 200 to 250 bp, and 250 to 300 bp including the adapters. These were used for 15 cycles of in-gel PCR. For the FOXA3 library 0.5 μg of immunoprecipitation material and 4 μg of input were used as the starting material. Here, three fragment sizes were also chosen for the immunoprecipitation material, but only the 250- to 300-bp fragment size was chosen from the input. The in-gel PCR was performed for 13 cycles. Amplified fragments were sequenced with 50-bp read lengths for FOXA1 and 35-bp read lengths for FOXA3. For H3K4me3, five immunoprecipitations from independent MNase-ChIP assays were pooled and used as the starting material. For this library preparation only one fragment size was chosen, as the sizes of fragments were uniform due to micrococcal nuclease treatment. The library was sequenced with a 35-bp read length.

### Co-immunoprecipitation

Cells were grown as described for ChIP. These were washed twice with cold 1× PBS, resuspended in a buffer containing 100 mM Tris-HCl (pH 8.0), 100 mM NaCl, 0.2% NP-40, and protease inhibitor cocktail for 30 minutes on ice and centrifuged in cold to collect nuclei in the pellet. The pellet containing intact nuclei was washed once with RIPA and resuspended in the same buffer, followed by homogenization in a Dounce homogenizer. Precleared nuclear lysate was incubated with antibody or IgG at a concentration of 1 μg per approximately 500 μg of total protein and 50 μl Protein G-agarose at 4°C overnight. Immune complexes were washed twice with RIPA buffer and eluted in NuPAGE^® ^LDS sample buffer (Invitrogen, Temecula, CA, USA) containing reducing agent (Invitrogen) at 70°C for 10 minutes. Samples were separated on NuPAGE^® ^4-12% for western blotting and detection with ECL detection system (GE Healthcare, Chalfont St Giles, UK). In order to verify the equal loading and as a negative control for the Co-IP experiments, one blot was stripped with 1× PBS, 0.1% Tween-20, and 0.2% SDS for 30 minutes at 65°C and incubated with GAPDH antibody (data not shown).

### Alignment, data management, and peak detection

ChIP-seq data were aligned using the SOLiD™ matching pipeline [36]. Three match errors in color space were allowed for the data sets with 35-bp reads (H3K4me3, FOXA3, and input). For the 50-bp reads in the FOXA1 experiment, four errors were allowed.

For FOXA1 and FOXA3, only reads starting at unique points were considered as those fragments were randomly sonicated and therefore expected to start at different positions. H3K4me3 ChIP-DNA had been sheared with MNase and the fragments were thus expected to be of defined lengths. Therefore, we did not merge H3K4me3 reads starting at the same position. Input reads were not merged either. Reads were then extended to represent the average fragment length in each of the samples (Table S1 in Additional data file 1) and genome-wide signals were created for reads on the forward strand, reads on the reverse strand, and overlapping fragments.

Peaks were then detected by first calculating a cutoff on the overlap signal. This cutoff was estimated in the following way. Assuming that a fragment of length *l*_*f *_is placed randomly onto a genome of length *l*_*g*_, the probability that one single base in the genome is covered by that fragment is *l*_*f*_/*l*_*g*_. If *n *reads are placed independently, the probability of having a certain number of overlaps at one position will follow the binomial distribution:

In this way, we can calculate *P*-values for observing at least *k *overlapping reads at any given position. These tests are then performed for each base in the genome. Because of the high number of reads in these experiments, and since the fraction *l*_*f*_/*l*_*g *_is very small, the binomial distribution can be effectively approximated by *Poisson (λ) *in this analysis where *λ = l*_*f*_/*l*_*g*_. So, in practice, this part of our peak finding method will yield the same results as other approaches that are based on the Poisson distribution [37]. Nevertheless, we chose to use the binomial distribution since it is a general and intuitive model for randomly placing millions of reads onto the genomic sequence. The Bonferroni method was used to correct for multiple hypothesis testing, a very stringent *P*-value correction.

Cutoffs were then obtained by selecting the lowest overlap value that gives a corrected *P*-value below 0.01: 15 for FOXA1, 21 for FOXA3, 15 for H3K4me3 and 16 for input. Peaks were defined by consecutive positions with overlap at least as high as the cutoff. In the next step, FOXA1, FOXA3, and H3K4me3 peaks that were also significantly enriched for input were removed. Examples of such regions are demonstrated in Figure S7 in Additional data file 1. High signals both in input and ChIP DNA are mostly found in regions close to centromeres where repeats are abundant. Such signals can arise when the reference genome has not been properly assembled. In the final step, we keep only regions with a significant number of overlapping forward reads upstream of the peak, or a significant number of reverse reads downstream. To take the forward and reverse reads into account is a commonly used strategy in ChIP-seq analysis [38,39]. We calculated cutoffs for forward and reverse peaks in the same way as for the overlap signal with *l*_*f *_now being the read length instead of fragment length as before. We allowed the forward or reverse peak to be at most a fragment length distance from the peak center. The reason why we do not require both a forward and a reverse peak is that repetitive elements immediately upstream or downstream of a peak can make it impossible to get uniquely aligned reads there.

### Motif search

We used the Bioconductor package BCRANK [21] for *de novo *motif searches in FOXA1 and FOXA3 data. BCRANK takes a ranked list as regions and works best on lists containing many regions, where the sequences at the bottom are not always bound directly by the TF. Therefore, we created ranked lists with FOXA1 and FOXA3 regions using more relaxed cutoffs than the ones described above by not applying the cutoffs for forward and reverse peaks. This resulted in 16,578 relaxed regions for FOXA1 and 22,066 for FOXA3.

### Annotations and Gene Ontology analysis

We annotated our regions to two types of transcripts. For well-characterized genes, we downloaded the UCSC Genes table from the UCSC Genome Browser [40], which contains predictions based on RefSeq, Genbank, CCDS, and UniProt. For less well-annotated transcripts, we downloaded the CAGE tag database from the FANTOM3 site [41], and extracted the 45,093 CAGE tag clusters found in HepG2 cells. In our analysis, all transcripts with a TSS within 1 kb of a peak are considered as bound by that factor. GO analysis was performed by applying the DAVID [42] functional annotation tools to sets of UCSC Genes. CpG island coordinates were downloaded from the UCSC Genome Browser.

### Expression analysis

Expression data were downloaded from Gene Expression Omnibus [43], accession number [GEO:GDS2213]. In the experiment, mRNA levels in HepG2 cells were measured in four replicates on the Affymetrix U133 Plus 2.0 array [44]. The expression level for each probe was calculated as the mean of the four replicates, and the probes were mapped to the corresponding UCSC Genes using the knownToU133Plus2 table from the UCSC Genome Browser. In this way, we obtained expression levels for 23,898 genes.

### Clustering of H3K4me3 patterns

For all 23,898 genes with expression levels in HepG2, we extracted the H3K4me3 signals in a 4-kb window surrounding the TSSs. The H3K4me3 signals were stored in numerical vectors with one value for each base in the window. k-means clustering was then applied to the 23,898 vectors. In this way, genes with similar H3K4me3 patterns around TSSs could be identified in an unsupervised manner. The same clustering strategy was performed for H3K4me3 signals at the 3'-end of the genes.

### Statistical analysis of allele-specific binding

Around 220,000 SNPs, heterozygous in HepG2, were identified by genotyping 1,000,000 SNPs on an Illumina array (data not shown). In the next step, we filtered out SNPs that were covered by at least ten reads. This resulted in three heterozygous SNPs for FOXA1, two for FOXA3, and six for H3K4me3. Among these heterozygous SNPs, we aimed to detect instances where ChIP-seq data were significantly biased towards one of the alleles. For this, we used SNP calling results from the SOLiD™ SNP calling pipeline [36] for each of H3K4me3, FOXA1, and FOXA3. We then assumed that both alleles should be equally represented if there is no allelic preference for the binding, and used a binomial model to test for the hypothesis. SNPs with a *P*-value < 0.001 were considered significant. To reach the significance threshold, a SNP must be covered by at least ten reads and, in that case, all reads must contain the same allele. For SNPs with even higher coverage, both alleles can be present but one significantly more often than the other. By multiplying the number of tested heterozygous SNPs (Table S8 in Additional data file 1) with the *P*-value significance threshold 0.001, we obtain the number of expected false positive allele-specific binding events for each factor. A comparison with the number of observations reveals that the false discovery rate is about 0.1 in our results, indicating that about one of the 11 identified SNPs in Table S8 in Additional data file 1 is expected to be a false positive.

### Data accession

The raw ChIP-seq data are freely accessible at the European Read Archive [45] with the accession number [ERA:000074]. The coordinates for our identified positive regions for FOXA1, FOXA3, and H3K4me3 are available as Additional data files 2, 3, and 4 and can be uploaded and viewed in the UCSC Genome Browser.

## Abbreviations

CAGE: cap-analysis of gene expression; ChIP: chromatin immunoprecipitation; ChIP-seq: ChIP with detection by sequencing; Co-IP: co-immunoprecipitation; FOX: Forkhead box/winged helix; GABP: GA binding protein; GO: Gene Ontology; H3K4me3: trimethylation of lysine 4 on histone H3; HNF: hepatocyte nuclear factor; MNase: micrococcal nuclease; PBS: phosphate-buffered saline; SNP: single nucleotide polymorphism; TF: transcription factor; TSS: transcriptional start site; USF: upstream stimulatory factor.

## Authors' contributions

CW and MM conceptualized the study; MM, MSRB, KP, MAB, and KM performed the experiments, library preparation, and the sequencing; AA, MM, JM, and JK conducted the analyses; OW provided data on FOXA2; MM, AA, and CW wrote the manuscript.

## Additional data files

The following additional data are available with the online version of this paper: a PDF file with supplementary results, materials and methods, Figures S1 to S8, and Tables S1 to S14 (Additional data file 1); a BED file with positions of FOXA1 in the HepG2 genome (Additional data file 2); a BED file with positions of FOXA3 in the HepG2 genome (Additional data file 3); a BED file with positions of H3K4me3 in the HepG2 genome (Additional data file 4); a text file containing data on heterozygous SNPs in HepG2 with position, ID, and alleles of each SNP (Additional data file 5).

## Supplementary Material

Additional data file 1 Supplementary results, materials and methods, Figures S1 to S8, and Tables S1 to S14. Click here for file

Additional data file 2 This file can be uploaded in the UCSC genome browser. Click here for file

Additional data file 3 This file can be uploaded in the UCSC genome browser. Click here for file

Additional data file 4 This file can be uploaded in the UCSC genome browser. Click here for file

Additional data file 5 This file can be viewed with MS Excel. Click here for file
