# Supplementary material for: Differential binding and co-binding pattern of FOXA1 and FOXA3 and their relation to H3K4me3 in HepG2 cells revealed by ChIP-seq
Source: Genome Biol. 2009 Nov 17;10(11):R129. doi: 10.1186/gb-2009-10-11-r129 (PMC3091322; doi:10.1186/gb-2009-10-11-r129)
Supplement: Additional data file 1 — Supplementary results, materials and methods, Figures S1 to S8, and Tables S1 to S14. [file gb-2009-10-11-r129-S1.PDF]

Supplementary Data

Supplementary Results

H3K4me3 at 3'-ends – antisense transcription

Transcriptional activity at the 3'-end of a gene in the opposite direction, could be indicative of antisense transcription. Thus, we performed the same type of clustering as before, this time dividing the signals into five clusters. A major part of these genes had no H3K4me3 at the 3'-end (Fig. S5). For two of the clusters, II and IV, we found enrichment for H3K4me3 downstream of the 3'-end, indicative of transcriptional activity in a tail-to-head manner. This was confirmed by comparison with the CAGE-tag data, where 39% (cluster II) and 41% (cluster IV) of the genes had CAGE-tags within 1 kb of their 3'-end indicative of a transcript on the same strand (Table S10). Although clusters II, IV, and V contained genes with evidence of antisense transcription at the 3'-end, these genes were relatively highly expressed and their 5'-ends contained H3K4me3 (Table S11 and S12). Thus, we found no clear support from our data that antisense transcription silences the transcription from the sense strand.

Verification of ChIP-seq regions

To verify the accuracy of our peaks, 35 and 47 putative regions with FOXA1 and FOXA3 binding were chosen, with number of overlaps both above and under the cut-off value, for validation with quantitative PCR (qPCR) (See Supplementary Methods). Enrichment for FOXA1 binding could be verified in 15 out of 19 regions. Among the supposedly negative regions, we could detect 2 false negatives out of 14 tested. For FOXA3, 19 out of 24 positive regions were verified, while 2 false negatives were found in the 21 tested.

For H3K4me3, 30 regions with enrichment and 9 without any enrichment were tested by qPCR, where enrichment or lack thereof could be verified to 100%. A portion of these primers was designed in a tiling manner covering 500 bp up- and downstream of the TSS of the *UROD* (uroporphyrinogen decarboxylase) gene. Comparison of the enrichment signals in this region for the ChIP-seq and the ChIP-qPCR demonstrated a good correlation (Fig. S8).

Supplementary Materials and methods

Verification by qPCR

For each of the factors at least four new ChIPs were performed, which were tested for enrichment before pooling. These were then used in qPCR with primers designed for both positive and negative regions (for primer sequences see Tables S13 and S14). For each primer a standard curve was obtained using the serial dilution of the input-DNA with which the C_t-value of the IP could be compared. Primers with double peaks or other abnormalities in the dissociation curve were disregarded. The standard curve was also used for calculation of primer efficiency, with the formula $10^{(-1/slope)}$, with which the quantity values for the IP were corrected. For each factor, five negative regions were tested. The quantitative values obtained for these regions were averaged and the standard deviation was calculated. These were then used to set a threshold value using the formula *Average + 2 x Standard deviation*. Tested primers with IP-values at least one fold over the threshold were considered as positive.

Supplementary Figures

Figure S1

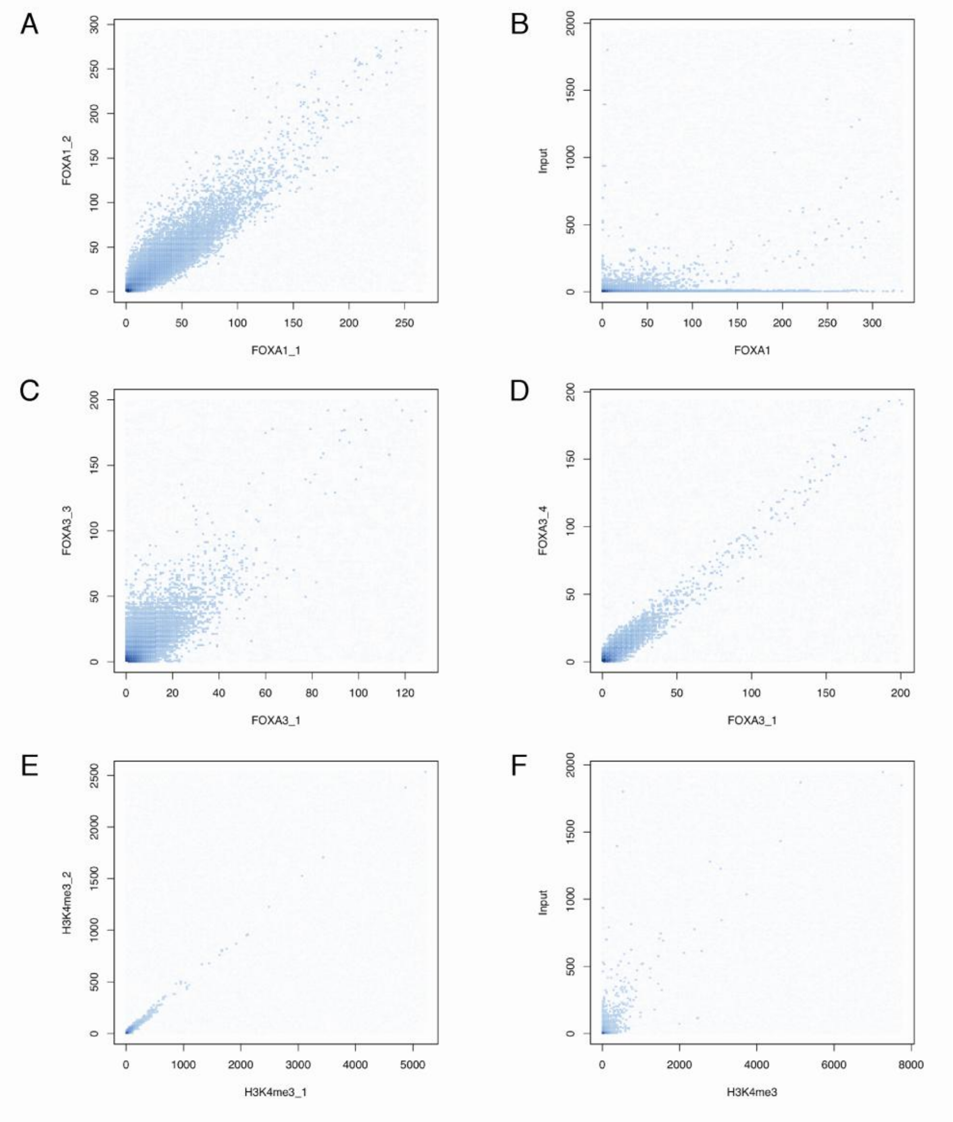

Figure S1. Correlations between ChIP-seq replicates and experiments.

The maximum overlap signal for unfiltered data in windows of 100 base pairs throughout the whole genome. For H3K4me3 and input multiple reads starting at the same position were not merged (see Materials and methods section), and therefore these have some regions with much higher overlap signals than FOXA1 and FOXA3. **(a)** Correlation between the two FOXA1 replicates. **(b)** FOXA1 versus input. **(c)** Correlation between two FOXA3 replicates with different fragment lengths. **(d)** Correlation between two FOXA3 replicates with the same fragment lengths. **(e)** Correlation between the two H3K4me3 replicates. **(f)** H3K4me3 versus input.

Figure S2

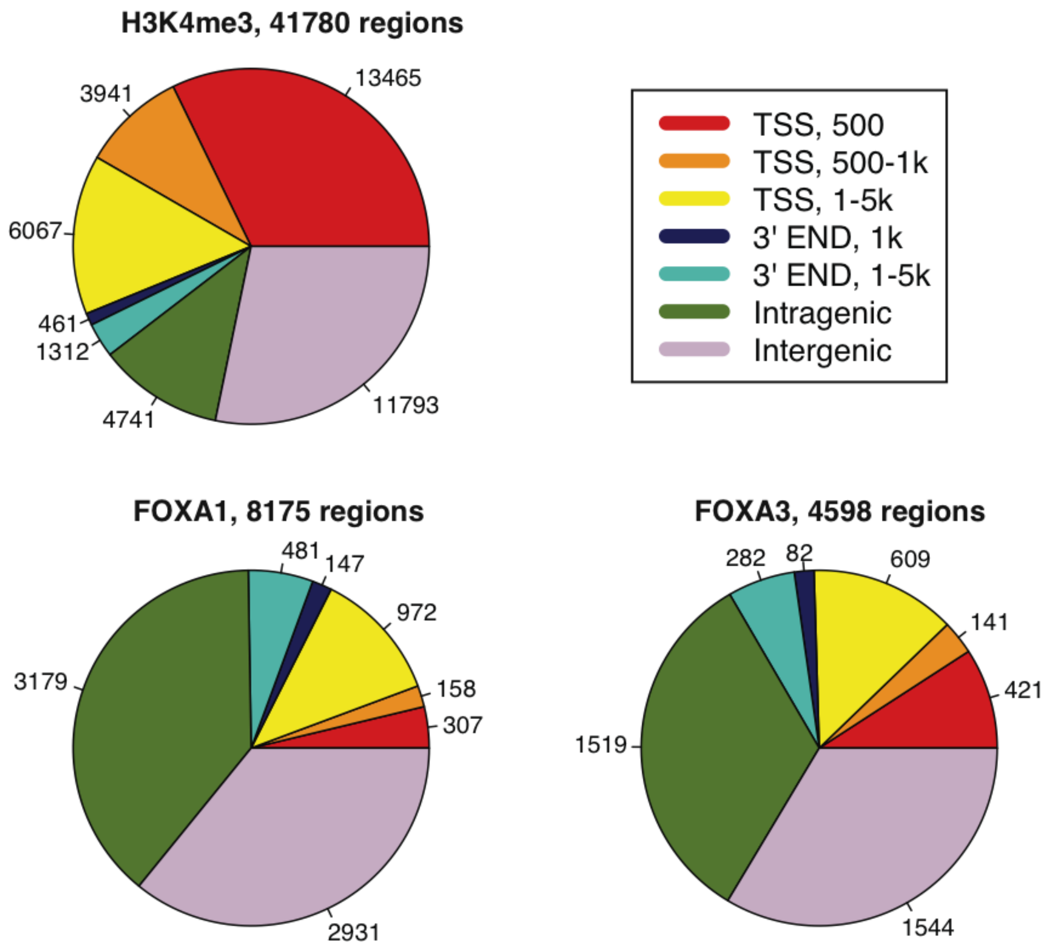

Figure S2. Genomic localization of the H3K4me3, FOXA1, and FOXA3 bound regions.

Each region was mapped to all UCSC gene coordinates and sequentially matched to the categories 500 bp from TSS, 1 kb from TSS, 5 kb from TSS, 1 kb from 3'-end, 5 kb from 3'-end and intragenic. The intergenic group consists of those regions not matching any of the mentioned categories.

Figure S3

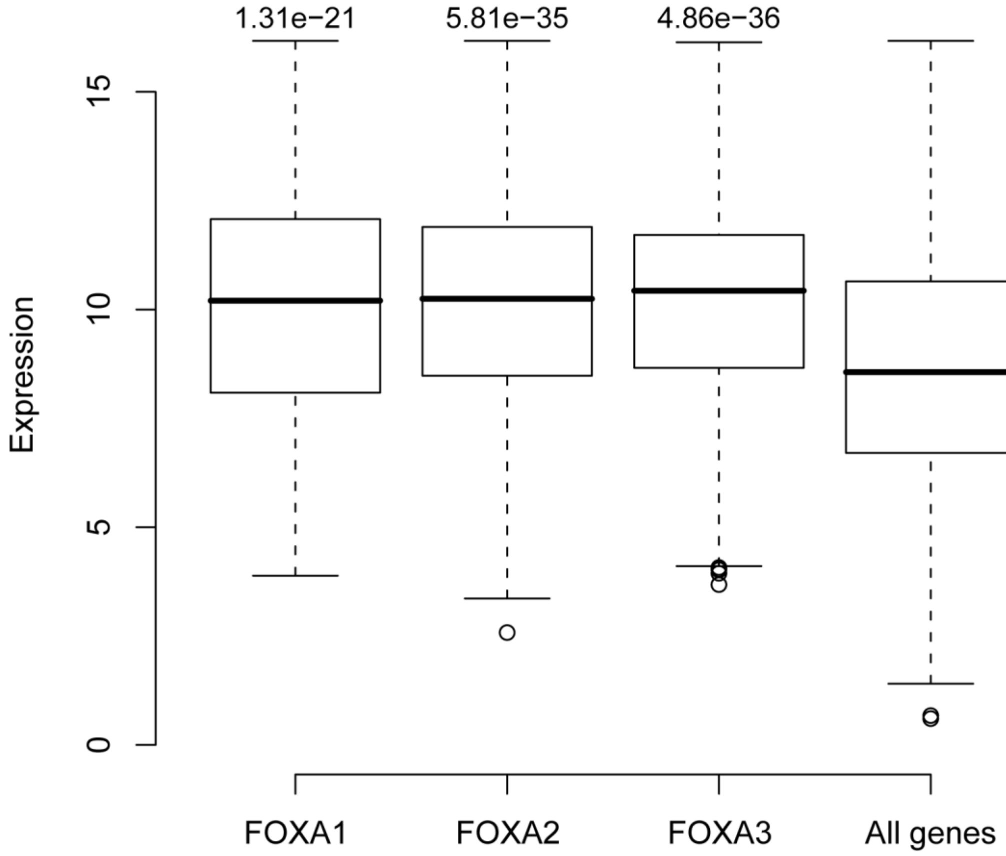

Figure S3. Expression of genes with a FOXA binding within 1 kb.

The box-plot shows the expression of genes with a FOXA binding site within 1 kb of the TSS of the genes. These are compared to expression of all genes in HepG2 cells. The p-values are given for comparison between each group and the total gene expression.

Figure S4

A)

Term	RT	Genes	Count	%	P-Value	Benjamini
multicellular organismal process	RT		132	28.6	6.6E-14	3.5E-10
anatomical structure development	RT		84	18.2	2.0E-10	5.1E-7
system development	RT		73	15.8	3.5E-10	6.2E-7
organ development	RT		59	12.8	6.9E-10	9.1E-7
multicellular organismal development	RT		84	18.2	1.3E-8	1.4E-5
positive regulation of biological process	RT		51	11.1	1.4E-8	1.2E-5
epidermis development	RT		16	3.5	1.8E-7	1.4E-4
ectoderm development	RT		16	3.5	4.6E-7	3.0E-4
positive regulation of cellular process	RT		44	9.5	5.7E-7	3.4E-4
developmental process	RT		98	21.3	2.6E-6	1.3E-3
tissue development	RT		22	4.8	2.9E-6	1.4E-3
positive regulation of transcription from RNA polymerase II promoter	RT		15	3.3	6.0E-6	2.6E-3
regulation of transcription from RNA polymerase II promoter	RT		26	5.6	6.2E-6	2.5E-3
system process	RT		53	11.5	1.1E-5	4.1E-3
response to wounding	RT		24	5.2	1.2E-5	4.0E-3
cell-cell signaling	RT		31	6.7	1.3E-5	4.3E-3
positive regulation of transcription, DNA-dependent	RT		18	3.9	1.5E-5	4.6E-3
sensory perception of light stimulus	RT		16	3.5	1.7E-5	4.8E-3
visual perception	RT		16	3.5	1.7E-5	4.8E-3
positive regulation of metabolic process	RT		24	5.2	1.9E-5	5.0E-3
positive regulation of cellular metabolic process	RT		23	5.0	2.1E-5	5.1E-3
response to stress	RT		42	9.1	2.6E-5	6.1E-3
[illegible]						

B)

Term	RT	Genes	Count	%	P-Value	Benjamini
multicellular organismal process	RT		141	25.3	1.7E-8	9.0E-5
developmental process	RT		121	21.7	6.8E-7	1.8E-3
response to external stimulus	RT		37	6.6	3.5E-6	6.1E-3
multicellular organismal development	RT		91	16.3	6.0E-6	7.8E-3
amine metabolic process	RT		28	5.0	2.5E-5	2.6E-2
positive regulation of biological process	RT		50	9.0	3.7E-5	3.2E-2
positive regulation of cellular process	RT		46	8.2	5.0E-5	3.7E-2
cell proliferation	RT		39	7.0	6.4E-5	4.1E-2
nitrogen compound metabolic process	RT		28	5.0	9.4E-5	5.3E-2
regulation of catalytic activity	RT		27	4.8	1.8E-4	8.9E-2
regulation of a molecular function	RT		29	5.2	2.4E-4	1.1E-1
system development	RT		66	11.8	4.5E-4	1.8E-1
positive regulation of programmed cell death	RT		17	3.0	5.9E-4	2.1E-1
death	RT		37	6.6	6.9E-4	2.3E-1
cell death	RT		37	6.6	6.9E-4	2.3E-1
regulation of kinase activity	RT		16	2.9	7.3E-4	2.1E-1
induction of programmed cell death	RT		15	2.7	8.2E-4	2.2E-1
blood circulation	RT		13	2.3	8.5E-4	2.1E-1
circulatory system process	RT		13	2.3	8.5E-4	2.1E-1
regulation of transferase activity	RT		16	2.9	9.0E-4	2.1E-1
regulation of programmed cell death	RT		27	4.8	9.2E-4	2.1E-1
response to wounding	RT		23	4.1	9.8E-4	2.1E-1
amino acid and derivative metabolic [illegible]	RT		21	3.8	1.0E-3	2.0E-1

Figure S4. The GO-categories for genes with FOXA1 or FOXA3 binding.

Genes with binding of FOXA1 **(a)** or FOXA3 **(b)** were categorized using Gene ontology.

Figure S5

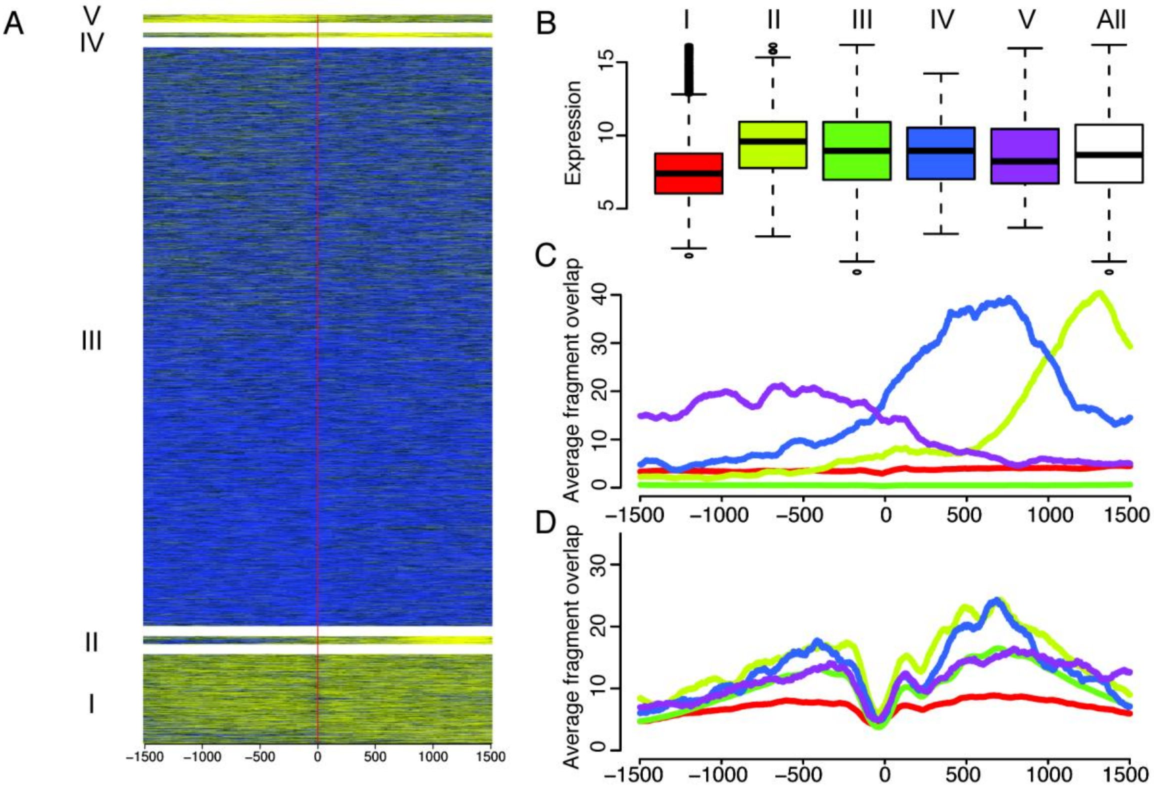

Figure S5. H3K4me3 signals around 3'-end of 20627 genes.

(a) Enrichment of H3K4me3 in a window surrounding the 3'-end. The genes were grouped into five clusters I-V by their H3K4me3 patterns. The enrichment scale is from high (yellow) to low (blue), and the red vertical line represents the 3'-end position. Negative x-coordinates are upstream of the 3'-end and positive are downstream. **(b)** Box plots with the distributions of expression levels in the five clusters. The white box represents the expression level for all genes. **(c)** Average H3K4me3 signal footprints for the five clusters at the 3'-end. The colors are as in **(b)**. **(d)** Average H3K4me3 signal footprints for the five clusters at the TSS. The colors are as in **(b)** and **(c)**.

Figure S6

A)

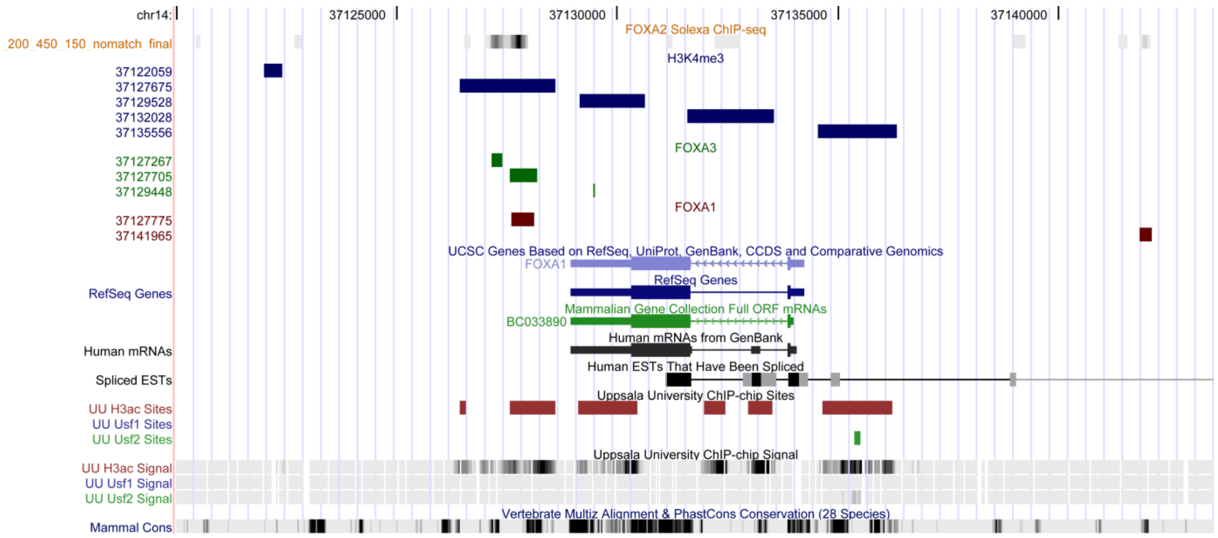

chr14:
37125000
37130000
37135000
37140000
FOXA2 Solexa ChIP-seq
200 450 150 nomatch final
H3K4me3
37122059
37127675
37129528
37132028
37135556
FOXA3
37127267
37127705
37129448
FOXA1
37127775
37141965
UCSC Genes Based on RefSeq, UniProt, GenBank, CCDS and Comparative Genomics
FOXA1
RefSeq Genes
Mammalian Gene Collection Full ORF mRNAs
BC033890
Human mRNAs from GenBank
Human mRNAs
Human ESTs That Have Been Spliced
Spliced ESTs
Uppsala University ChIP-chip Sites
UU H3ac Sites
UU Usf1 Sites
UU Usf2 Sites
Uppsala University ChIP-chip Signal
UU H3ac Signal
UU Usf1 Signal
UU Usf2 Signal
Vertebrate Multiz Alignment & PhastCons Conservation (28 Species)
Mammal Cons

B)

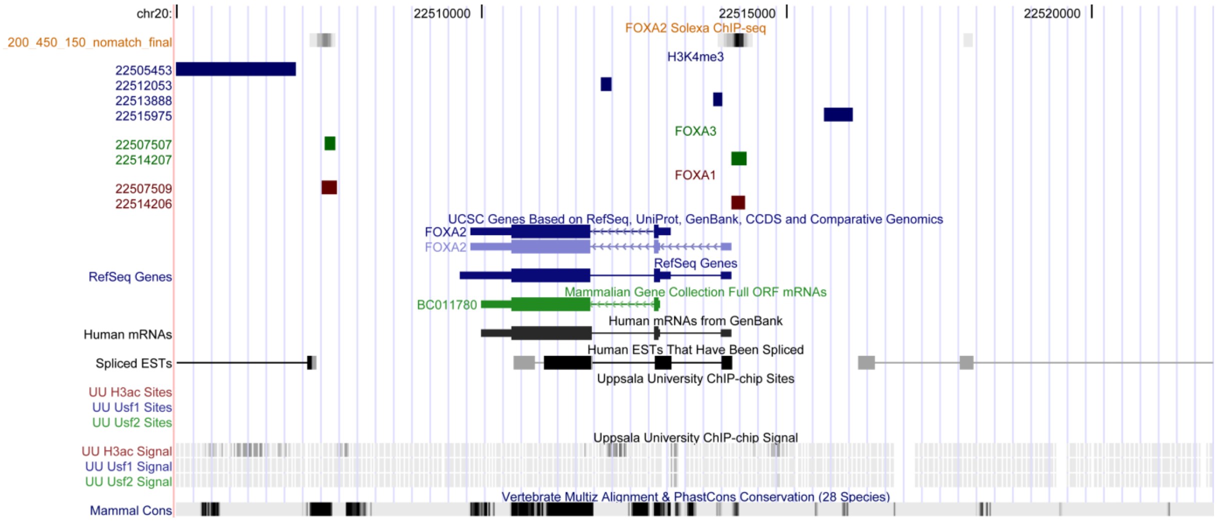

chr20:
22510000
22515000
22520000
FOXA2 Solexa ChIP-seq
200 450 150 nomatch final
H3K4me3
22505453
22512053
22513888
22515975
FOXA3
22507507
22514207
FOXA1
22507509
22514206
UCSC Genes Based on RefSeq, UniProt, GenBank, CCDS and Comparative Genomics
FOXA2
FOXA2
RefSeq Genes
Mammalian Gene Collection Full ORF mRNAs
BC011780
Human mRNAs from GenBank
Human mRNAs
Human ESTs That Have Been Spliced
Spliced ESTs
Uppsala University ChIP-chip Sites
UU H3ac Sites
UU Usf1 Sites
UU Usf2 Sites
Uppsala University ChIP-chip Signal
UU H3ac Signal
UU Usf1 Signal
UU Usf2 Signal
Vertebrate Multiz Alignment & PhastCons Conservation (28 Species)
Mammal Cons

C)

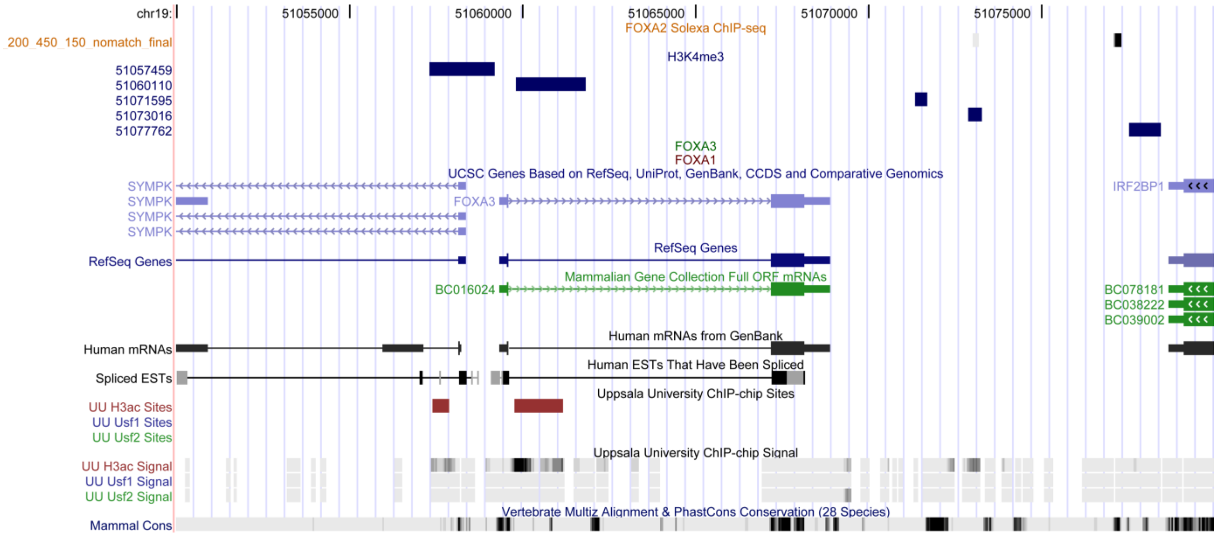

Figure S6. UCSC genome browser window over FOXA genes.

(a) Human *FOXA1* on chromosome 14, **(b)** *FOXA2* on chromosome 20, and **(c)** *FOXA3* on chromosome 19, showing binding sites of FOXA1, FOXA2, and FOXA3 together with H3K4me3.

Figure S7

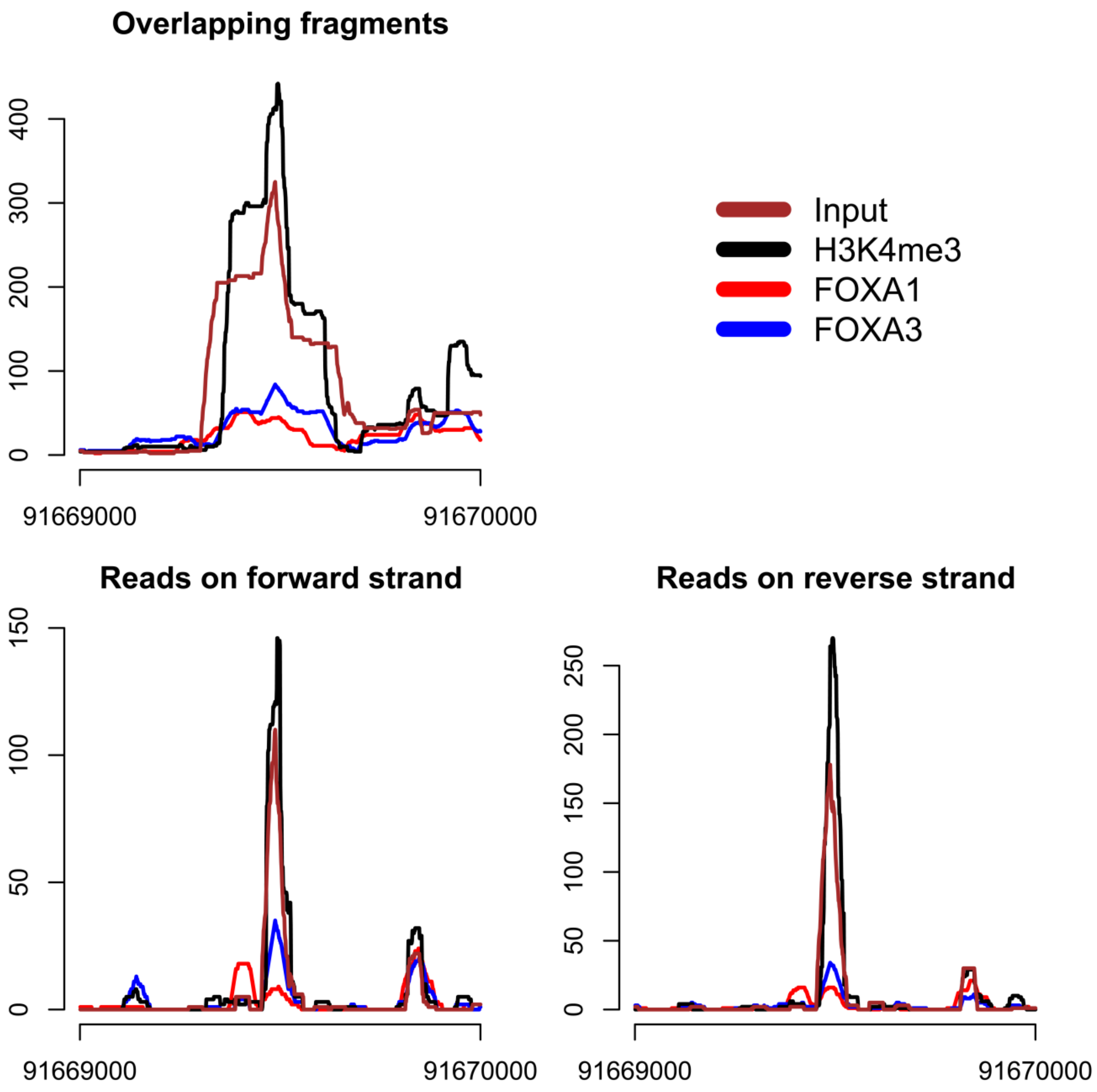

Figure S7. Examples of regions with signal in the sequenced input DNA.

The panel above shows an example of a region with signal in input, while the two lower panels show the signals separated for the forward and reverse reads.

Figure S8

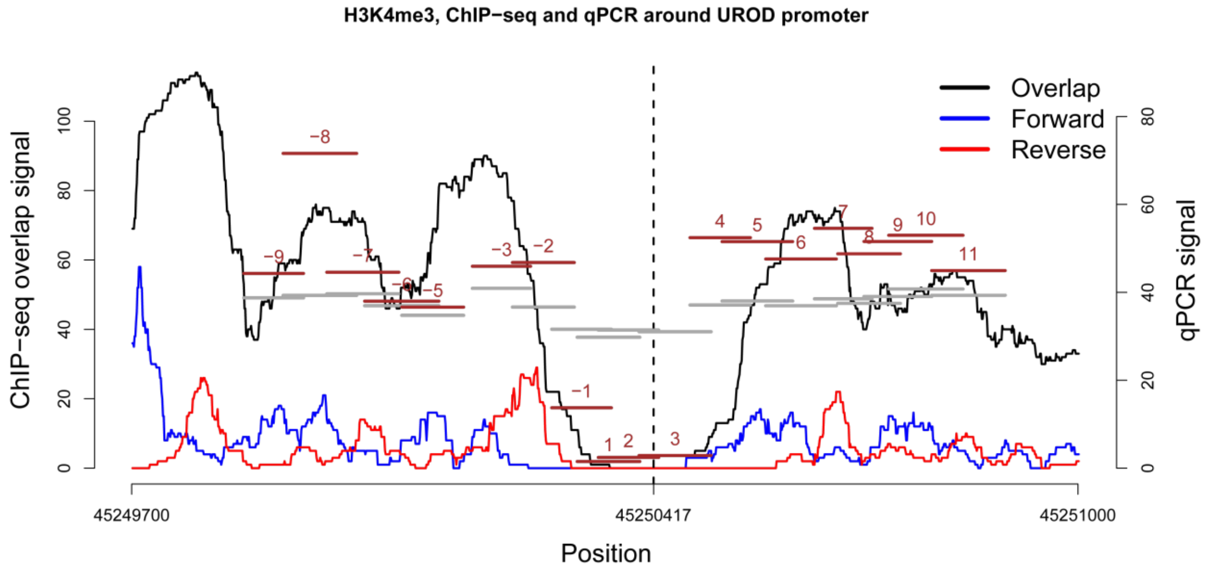

Figure S8. Correlation of enrichment in ChIP-seq and ChIP-qPCR.

Primers were designed to cover 500 bp up- and downstream of the TSS of the *UROD* gene. These primers were then used in qPCR for verification of the enrichment signals from the ChIP-seq of H3K4me3. The vertical hatched line indicates the TSS of *UROD*. Blue and red curves are reads from the forward and reverse strand, respectively, while the black indicates the average signal obtained for H3K4me3 ChIP-seq enrichment in this region. Numbered brown lines are the tiling primers with the hight in the graph indicating the level of enrichment in qPCR. The gray lines specify the signal for the same sequence amplified by qPCR in the input material (*i.e*. no enrichment/total genomic DNA).

Supplementary Tables

Table S1. Summary data for ChIP-seq regions.

	FOXA1	FOXA3	H3K4me3	Input
Read length	50 bp	35 bp	35 bp	35 bp
Total reads	51.9 M	166.3 M	58.1 M	53.5 M
Aligned reads	20 M	55.3 M	24.5 M	20.2 M
Unique starts	18.6 M	49.7 M	-	-
Length of extended fragments	186	154	147	187
Overlap cut-off	15	21	15	16
Distance between regions	186	154	400	187
Input cut-off	16	16	16	-
Fwd/rev cut-off	9/9	10/10	8/8	-
Number of peaks	8,175	4,598	41,780	-
Average peak lengths	296 bp	260 bp	764 bp	-

Table S2. P-values for expression differences in Fig. 2b (two-tailed *t*-test).

	Cluster II	Cluster III	Cluster IV	Cluster V	Cluster VI	Cluster VII	All genes
Cluster I	5.5e-26	2.48e-17	1.38e-18	2.84e-178	9.17e-65	1.33e-06	1.66e-110
Cluster II		0.00673	0.00334	2.02e-200	8.76e-15	2.94e-16	2.05e-70
Cluster III			0.964	1.91e-240	7.47e-29	1e-07	1.48e-100
Cluster IV				1.98e-314	2.76e-35	9.76e-09	2.28e-131
Cluster V					< 10e-325	< 10e-325	< 10e-325
Cluster VI						5.39e-84	1.2e-53
Cluster VII							3.12e-228

Table S3. Properties of different clusters of H3K4me3 at TSS of known genes.

	# TSSs	Bidirectional CAGE-tag within 1kb	FOXA1 ±1k	FOXA3 ±1k
Cluster I	596	237 (40%)	34 (5.7%)	67 (11.2%)
Cluster II	1,406	854 (61%)	37 (2.6%)	63 (4.5%)
Cluster III	1,361	455 (33%)	26 (1.9%)	46 (3.4%)
Cluster IV	1,732	422 (24%)	59 (3.4%)	87 (5.0%)
Cluster V	12,776	649 (5%)	89 (0.7%)	71 (0.6%)
Cluster VI	4,043	895 (22%)	92 (2.3%)	111 (2.7%)
Cluster VII	1,935	590 (30%)	41 (2.1%)	80 (4.1%)
Total	23,849	4,102 (17%)	378 (1.6%)	525 (2.2%)

Table S4. Top 3 GO-categories of the different H3K4me3 clusters at TSS.

	First GO-term	Second GO-term	Third GO-term
Cluster I	establishment of protein localization 5.3E-6	protein transport 5.8E-6	protein localization 6.3E-6
Cluster II	cellular metabolic process 2.4E-13	metabolic process 5.8E-13	primary metabolic process 7.8E-13
Cluster III	metabolic process 1.7E-12	cellular metabolic process 3.8E-12	macromolecule metabolic process 9.3E-11
Cluster IV	metabolic process 1.6E-24	cellular metabolic process 2.5E-22	primary metabolic process 1.3E-18
Cluster V	multicellular organismal process 6.6E-121	multicellular organismal development 1.0E-102	developmental process 6.3E-87
Cluster VI	primary metabolic process 5.6E-20	regulation of biological process 9.2E-20	biological regulation 3.4E-19
Cluster VII	primary metabolic process 3.3E-17	macromolecule metabolic process 6.8E-16	metabolic process 3.6E-15

Table S5. Sequence elements at TSS of known genes for the different clusters of H3K4me3.

	# TSSs	CpG ±1 kb	TATA ±50 bp	CAAT ±50 bp
Cluster I	596	545 (91%)	20 (3%)	178 (30%)
Cluster II	1,406	1,303 (93%)	74 (5%)	415 (30%)
Cluster III	1,361	1,237 (91%)	78 (6%)	351 (26%)
Cluster IV	1,732	1,415 (82%)	106 (6%)	578 (33%)
Cluster V	12,776	3,964 (31%)	2,780 (22%)	5,989 (47%)
Cluster VI	4,043	3,394 (83%)	278 (7%)	1,023 (25%)
Cluster VII	1,935	1,856 (96%)	95 (5%)	427 (22%)
Total	23,849	7,551 (57%)	3,431 (14%)	8,961 (38%)

Table S6. Number of TSSs in regions with FOXA1-2-3 binding.

	# FOXA1-2-3 regions	Fwd TSS (5 kb)	Rev TSS (5 kb)
Cluster I	116	57 (49%)	38 (33%)
Cluster II	141	50 (35%)	58 (41%)
Cluster III	493	49 (10%)	71 (14%)
Cluster IV	1,553	116 (7%)	109 (7%)
Total	2,304	272 (12%)	276 (12%)

Table S7. Number of CAGE-tags in regions with FOXA1-2-3 binding.

	# FOXA1-2-3 regions	Fwd CAGE (5 kb)	Rev CAGE (5 kb)	Fwd CAGE (1 kb)	Rev CAGE (1 kb)
Cluster I	116	73 (63%)	47 (41%)	50 (43%)	22 (19%)
Cluster II	141	59 (42%)	73 (52%)	26 (18%)	48 (34%)
Cluster III	493	82 (17%)	83 (17%)	40 (8%)	27 (5%)
Cluster IV	1,553	141 (9%)	142 (9%)	31 (2%)	32 (2%)
Total	2,304	355 (15%)	345 (15%)	147 (6%)	129 (6%)

Table S8. SNPs found by sequencing in the ChIP-seq data.

**FOXA1**	**Tested hz SNPs: 145, FDR: 0.07**				
Chr	*Position*	*ID*	*Ref read*	*Other read*	*P-value*
Chr6	34,064,143	rs2499767	A (28) [a]	G (58)	0.0008027825
Chr19	7,175,431	rs7248104	G (0)	A (15)	3.051758e-05
Chr2	43,060,485	rs921068	A (49)	G (18)	9.71489e-05
**FOXA3**	**Tested hz SNPs: 75, FDR: 0.04**				
Chr	*Position*	*ID*	*Ref read*	*Other read*	*P-value*
Chr2	19,015,666	rs2271065	A (0)[a]	G (14)	6.103516e-05
Chr6	159,140,285	rs3127186	T (0)	C (11)	0.0004882812
**H3K4me3**	**Tested hz SNPs: 715, FDR: 0.12**				
Chr	*Position*	*ID*	*Ref read*	*Other read*	*P-value*
Chr6	26,231,518	rs198822 [b]	G (0)	C (12)	0.0002441406
Chr9	79,239,118	rs1801258	C (0)	T (16)	1.525879e-05
Chr10	124,628,817	rs4638251	G (0)	A (10)	0.0009765625
Chr14	31,743,179	rs17098557	A (9)	G (31)	0.0003397741
Chr2	206,731,840	rs4147709	C (27)	M (8)	0.0009391127
Chr10	5,126,651	rs12529	C (53)	G (21)	0.0001282443

[a] The number of reads for each of the alleles is indicated in the parenthesis.

[b] This SNP was found on reads from one strand only.

Table S9. Comparison of ChIP-seq data with genome-wide association studies for identification of functional SNPs.

	Chr	Position	SNP	Nearest/affected gene	Factor	Trait	Study
	1	25,631,242	rs873308	*TMEM57*	H3K4me3	TC[b]	Aulchenko et al
*[a]	1	109,619,113	rs12740374	*CELSR2, PSRC1, SORT1*	FOXA1	LDL[c]/Dyslipidemia	Kathiresan et al
	1	109,619,829	rs629301	*SARS/CELSR2/MYBPHL*	H3K4me3	LDL	Aulchenko et al
*	1	109,620,053	rs646776	*CELSR2-PSRC1-SORT1*	H3K4me3	TC/LDL	Aulchenko et al, Sabatti et al
	1	163,866,770	rs4147592	*MTNR1B*	H3K4me3	Fasting glucose levels	Prokopenko et al
	2	21,232,310	rs312985	*APOB*	FOXA1	TC	Aulchenko et al
*	2	43,918,594	rs6756629	*ABCG5*	H3K4me3	TC/LDL	Aulchenko et al
	2	213,812,024	rs10497997	*MTNR1B*	FOXA1	Fasting glucose levels	Prokopenko et al
	5	74,667,889	rs3761740	*GCNT4/HMGCR/POLK*	H3K4me3	TC	Aulchenko et al
	5	75,000,878	rs34358	*GCNT4/HMGCR/POLK*	H3K4me3	TC	Aulchenko et al
	9	106,688,473	rs3847303	*ABCA1*	FOXA1	HDL[d]	Aulchenko et al
	10	101,835,424	rs11596076	*CPN1*	FOXA1	Plasma levels of liver enzymes (ALT[e])	Yuan et al
*	10	101,851,425	rs11597390	*CPN1*	FOXA1, FOXA3	Plasma levels of liver enzymes (ALT)	Yuan et al
*	11	47,226,831	rs2167079	*NR1H3*	H3K4me3	HDL	Sabatti et al
*	11	61,353,788	rs174570	*FADS2/3*	H3K4me3	TC/LDL	Aulchenko et al
	11	116,167,617	rs3135506	*BUD13/APO(A1/A4/A5/C3)*	H3K4me3	TG[f]	Aulchenko et al
	11	116,448,564	rs1351452	*BUD13/APO(A1/A4/A5/C3)*	FOXA1	TG	Aulchenko et al
	15	56,510,967	rs1800588	*LIPC*	H3K4me3	HDL	Sabatti et al
	16	55,526,649	rs2217332	*NUP93/HERPUD1/CETP*	H3K4me3	HDL	Aulchenko et al
*	19	11,063,306	rs6511720	*LDLR*	H3K4me3	TC/LDL/Dyslipidemia	Aulchenko et al, Sabatti et al, Kathiresan et al
*	19	50,087,106	rs157580	*TOMM40-APOE, APO* cluster	H3K4me3	TC/TG/LDL	Aulchenko et al, Sabatti et al
*	19	50,087,459	rs2075650	*TOMM40-APOE*	H3K4me3	CRP[g]/TC/LDL	Sabatti et al, Aulchenko et al
	19	50,100,676	rs405509	*CEACAM16 - TOMM40*	H3K4me3	LDL	Aulchenko et al

	Chr	Position	SNP	Nearest/affected gene	Factor	Trait	Study
	19	60,359,618	rs3729709	*MTNR1B*	H3K4me3	Fasting glucose levels	Prokopenko et al
*	22	23,320,213	rs4820599	*GGT1*	H3K4me3	Plasma levels of liver enzymes (GGT[h])	Yuan et al

[a] These SNPs are presented in Table 3.

[b] TC – Total cholestrol

[c] LDL – Low density lipoproteins

[d] HDL – High density lipoproteins

[e] ALT – Alanine-aminotransferase

[f] TG – Triglycerides

[g] CRP – Indicator of inflammation

[h] GGT – gamma-glutamyl transferase

Table S10. Properties of different clusters of H3K4me3 at 3'-end of known genes.

	# 3'-ends	CAGE-tag, same 1 k	CAGE-tag, opp 1 k	CpG, 1 k	FOXA1, 1 k	FOXA3, 1 k
Cluster I	2,685	229 (8.5%)	70 (2.6%)	380 (14.2%)	68 (2.5%)	36 (1.3%)
Cluster II	243	94 (38.6%)	21 (8.6%)	101 (41.6%)	5 (2.1%)	7 (2.8%)
Cluster III	17,317	1,477 (8.5%)	302 (1.7%)	1,018 (5.9%)	69 (0.4%)	46 (0.3%)
Cluster IV	140	58 (41.4%)	25 (17.8%)	71 (50.7%)	6 (4.3%)	8 (5.7%)
Cluster V	242	69 (28.5%)	23 (9.5%)	90 (37.2%)	20 (8.2%)	15 (6.2%)
**Total**	**20,627**	**1,927 (9.3%)**	**441 (2.1%)**	**1,660 (8%)**	**168 (0.8%)**	**112 (0.5%)**

Table S11. Top 3 GO-categories of the different H3K4me3 clusters at 3'-ends.

	First GO-term	Second GO-term	Third GO-term
Cluster I	anatomical structure development 9.8E-26	system development 6.3E-26	organ development 6.7E-22
Cluster II	organelle organization and biogenesis 1.0E0	macromolecule biosynthetic process 1.0E0	mitochondrion organization and biogenesis 1.0E0
Cluster III	primary metabolic process 2.2E-45	metabolic process 2.7E-38	cellular metabolic process 2.9E-38
Cluster IV	positive regulation of cellular process 5.5E-1	positive regulation of biological process 3.3E-1	positive regulation of transcription 6.2E-1
Cluster V	negative regulation of cellular process 9.9E-1	negative regulation of biological process 9.9E-1	Phototransduction 9.7E-1

Table S12. P-values for expression differences in Fig S5b (two-tailed *t*-test).

	Cluster II	Cluster III	Cluster IV	Cluster V	All genes
Cluster I	9.25e-25	3.65e-141	1.52e-06	9.89e-09	5.25e-112
Cluster II		0.000863	0.00328	0.00224	1.31e-05
Cluster III			0.251	0.263	3.82e-10
Cluster IV				0.893	0.727
Cluster V					0.843

Table S13. Primer sequences for verification of FOXA1 and FOXA3 ChIP-seq by qPCR.

#	Primer name	Forward primer	Reverse primer	Primer coordinates
1	FOXA3-100-242	GTCCAAGGGGTGCTGTTTAC	GGCTGACTGTGCAGAAACAT	>chr20:25436865+25436943
2	FOXA3-101-57	GCACATCCACTCTGCAAGG	CCACCGTTCCTGTCTTGTTT	>chr22:35077320+35077398
3	FOXA3-102-39	TGCACAACCCACACTTAACC	TGTCCTGTCACTGGATCTGG	>chr1:224140662+224140740
4	FOXA3-103-32	TCCACACCCACATGTTGACT	CATTTGCTCCCAGGTGATTT	>chr6:35241643+35241721
5	FOXA3-104-28	AGCTTTTCCATTAGGACTGTGA	GCTACGGAGACCAAGTAGGC	>chr2:9714748+9714853
6	FOXA3-105-26	GGGATGCTCAGGAATGCTT	CCAGCTGACCTCCCTAGGAT	>chr9:67903095+67903194
7	FOXA3-106-25	TGGTGCTTGGATGTTTGTGT	ACACATCTGGGACGCATTTC	>chr20:35468137+35468216
8	FOXA3-107-23	AAATGAACCTCCGTTGTTGG	CCATGTCTTGCTTTCCTTTTC	>chr2:8430441+8430525
9	FOXA3-108-23	GCTCCAGACACTCTGGCTTC	GGAACAGCAAAGGCAAAGG	>chr20:44770855+44770938
10	FOXA3-109-22	TATGTGGCATGGACTGTTGC	TAGGTTTGTGGGACCTGCTT	>chr14:33781512+33781593
11	FOXA3-110-21	TCTTGAGTCACATGGGATCTTTT	CCCGAGGGCATGTAAACTAA	>chr6:32252880+32252959
12	FOXA3-111-81	AGGGCAGGGACCATCTATCT	GCGCCCTCTAGTGGCTTAGT	>chr21:36435720+36435811
13	FOXA3-112-49	CCTACAGTGGCCTTGTCCAT	TGCTAGGAATTGGGATGTCA	>chr17:34030634+34030710
14	FOXA3-114-46	GTGTGGAACTCCAGGCAAC	GGCCAATGTAAACATGACCA	>chr7:99472313+99472387
15	FOXA3-115-51	GCAGGACACTGTGGTCTGGT	TATACCCTGAGTGGCTTTGG	>chr19:1822499+1822595
16	FOXA3-116-54	GTGGACAAAGTCCCTGCTGT	TGCCCCATCCTGTGTTTATT	>chr5:149882389+149882482
17	FOXA3-117-59	GCTGGACTTTGACACCCATT	GACCCTCCCTAGCCACTTTC	>chr14:49306951+49307030
18	FOXA3-118-63	GGAAGACAGGGGTCAATGAA	TTTGTGACCAATTATGAGTTGCTT	>chr20:51629722+51629803
19	FOXA3-119-66	ATTTGAAGGCCAAGCTCAGA	CTTGTGGCCACGTGACTAAG	>chr20:45243466+45243548
20	FOXA3-120-71	TTAATCTGCTGCCCTCCTGT	TGCAATGAACCATAGGCTTG	>chr2:38516961+38517035
21	FOXA3-121-76	TTGTTGAATAATATTTGCTGTGGAG	CGGGAAAACCTTCCACTTC	>chr10:106038306+106038402
22	FOXA3-122-83	TGAAGTGGGTCTGCTATCACC	CTTGGGCTCAGTGGAAATTG	>chr2:64811272+64811361
23	FOXA3-123-90	TCTCTGTGGACATGATTGGTG	TCTACAGAGAGTGTCTGATGTTTGC	>chr10:91123224+91123298

#	Primer name	Forward primer	Reverse primer	Primer coordinates
24	FOXA3-124-104	AACCACTGCTAAAGACTGGTCA	CGTCCCTTAGTCAGGCATGT	>chr5:38585869+38585959
25	FOXA3-125-121	CAAAGGGAAACCAGTCAAGG	TGCTTTTGTGATAATTACACCTATTCT	>chr3:81432384+81432468
26	FOXA1-132-333	TTAGACCAGACACCTCAGTCCA	TCTGTTGCTTAATGTCACCTACTG	>chr10:53882571+53882645
27	FOXA1-133-102	GCACCCACAGTAGATCAAAGG	CAAAGGAATGTGGGGAGTTT	>chr12:15660905+15660984
28	FOXA1-134-73	AGAGGATTGCACAACCTTTG	GCAACGCCTGTCACATTTTA	>chr2:200311871+200311958
29	FOXA1-135-59	TCCTGAGCAGCTGGTACTCC	CCACAAGTGTGTTTACTCAAAGC	>chr22:21071458+21071533
30	FOXA1-136-49	CCGGCTAGAGAGAGACACGA	AATCAGCTGGCACACACTTG	>chr17:58863608+58863705
31	FOXA1-137-42	TTGACAGACACTGTGTTTCTTCTG	CCAGCTGGGGTGTTATTCTG	>chr18:54370545+54370635
32	FOXA1-138-36	GGGGGTTGGATCACATCATA	TAGCGCATTGCAAAATCAAG	>chr2:120822617+120822694
33	FOXA1-139-32	AAAGTGGTCTGTGGCTTCAAA	ATTCAAAAGGCACACAAGCA	>chr2:205847931+205848009
34	FOXA1-140-29	TTGGCCTGTCCTATGGTTTG	AAGGCATTCCATGTCTCCAG	>chr6:142669723+142669817
35	FOXA1-141-26	TGACCTCTCACCGCCTTCTA	GTCAAGCAAACAAACCGTGA	>chr1:166929121+166929199
36	FOXA1-142-24	CCAAATTCCTTTCCCTCTGG	TGGAATAGAGCCATAGCTTCC	>chr2:192427521+192427599
37	FOXA1-143-23	TCGCTTTTAAGTGACAGTAGTTGTG	AGAGAGAGATCAAAGGGATTCG	>chr20:5760303+5760382
38	FOXA1-144-21	CCCCTGAGTCCCAGTAGTGA	ATGTTGCATCACCTCCCTGT	>chr8:136620588+136620679
39	FOXA1-100-213-APO	GCTCTCTGCCTTCCAGAATC	GGGCACAAGGTAGGTCCATA	>chr11:116184233+116184315
40	FOXA1-101-49-APO	GTCCTCAGGCTTGCCACTAC	CATCTAGCAGTGGGGCATTT	>chr11:116184885+116184965
41	FOXA1-102-31-APO	GCTGCCCCAGTAGAGAAGTG	TTAAAGGTCATGGGGTTTGG	>chr11:116168495+116168585
42	FOXA1-103-135-APO	CCTGTCACTTTGTGCCTCAG	AACCCCAGCACAAGTCAAAC	>chr11:116208410+116208491
43	FOXA1-105-412	TAAAATGCCTTCCGGACAAC	CTGTCTGGCTGCTTTGATCC	>chr2:101549739+101549829
44	FOXA1-108-57	CTGGACCAGGGTAATGAGGA	CACAGGTGTGTGCAACAAAA	>chr5:171743171+171743248
45	FOXA1-109-47	CCAAACACCGGCAAAACTAT	TGTGGCAGGGGAGCTAGTTA	>chr6:154823561+154823636
46	FOXA1-111-31	CATCGGCAAGGTGTCTTTTT	ACCAAAGGGCATGCATAACT	>chr6:151432815+151432902
47	FOXA1-129-103	TCAGCTGATGACAGACTTTTAATC	CTGTGTGTCAGGTACATCTGTGA	>chr14:74482259+74482348

Table S14. Primer sequences for verification of H3K4me3 ChIP-seq by qPCR.

#	Primer Name	Forward primer	Reverse primer	Primer coordinates
1	UROD_-1	TTTACAGCGGAGTTTTCTTTTG	CCGTAGGACCCCTACCTTTT	>chr1:45250277+45250359
2	UROD_-2	CGGATACCCAGACTGTCAGA	AACAAAGGCCCAAAAGAAAA	>chr1:45250223+45250308
3	UROD_-3	TGGACCTGGCTGGATAAGAC	TTCTCATCTGACAGTCTGGGTATC	>chr1:45250168+45250248
4	UROD_-4	TTAAAGGCTGGGGCTGTCTT	TCTTATCCAGCCAGGTCCAT	>chr1:45250103+45250186
5	UROD_-5	GCTGATTTCTGTTTCCCCAGT	GGACCTATAGTAGGTGCAGAGGTT	>chr1:45250071+45250156
6	UROD_-6	TCACTCCTAATCCCAAGGACA	AAGACAGCCCCAGCCTTTA	>chr1:45250020+45250122
7	UROD_-7	CCTTCCCCTGCCACTGAC	CACATTAAAATGAATAATGATCTCCA	>chr1:45249968+45250067
8	UROD_-8	CGAATCTGCTGGACTCCCTA	CAACCCTAATGACTCAAGACTCC	>chr1:45249908+45250009
9	UROD_-9	GCCAACAGTCATTTTTCTGG	CCCATAGGATAGGGAGTCCA	>chr1:45249854+45249936
10	UROD_1	GCGTCGCTACAGCAAACTTTA	GGACTCCAGCGACAGCTC	>chr1:45250312+45250398
11	UROD_2	AAAGGTAGGGGTCCTACGG	GTCGCCATGTTGAAGATCAC	>chr1:45250341+45250424
12	UROD_3	CCGATCATGTGATCTTCAACA	TCCACAATTTAACCTGAATCTGA	>chr1:45250397+45250496
13	UROD_4	GGCAGGCTCAGATTCAGGT	TCTGGAGAACTCACCCCAAC	>chr1:45250467+45250558
14	UROD_5	ACAGACAGCTGACCATGGAA	TCCTGAGTTGGAAGACTCAAA	>chr1:45250511+45250608
15	UROD_6	GCTAGCCGGGCTTCTAATTT	GCTAACGGAGTTCAGGTTGG	>chr1:45250571+45250668
16	UROD_7	CTGGAGACCTCCCAACCTG	CCTGGGATGAACATCTCAAAA	>chr1:45250638+45250717
17	UROD_8	GGGATCCTGAATCCTAAAACC	TCCTGGTTGGAAATCCTGAG	>chr1:45250670+45250756
18	UROD_9	GTTCATCCCAGGGCCTTAAT	GGGGGATAAAGAGGGATCAG	>chr1:45250706+45250799
19	UROD_10	AGGATTTCCAACCAGGATCTC	GAATGTCACTGGGCTGGTTC	>chr1:45250740+45250842
20	UROD_11	CAGCCTGGGTATTTCTCAGC	AATGAAGGCCCACTAAGGAT	>chr1:45250799+45250900
21	HMG20A 5	TTTTGTCCAGCTGTGAGACG	GCCAAAAGAAAGTCCCTTCC	>chr15:75500277+75500369
22	HMG20A 1	TGGGGCAAAATAATCCCTTC	TTTACAATTGCTCCGGTGAG	>chr15:75500103+75500182
23	HMG20A 8	GTGGCCCCAGTCGAGATG	GCCTCCAACTGAGACAAACC	>chr15:75500427+75500510

#	Primer Name	Forward primer	Reverse primer	Primer coordinates
24	RPL27_1	GAAGCTTGGGTTGAATCTTTC	CGCCAGAGACCTAGGGAAG	>chr17:38404022+38404118
25	RPL27_7	GGAAGGTGGTGCTTGTCCT	CATGCAAGGACGCAGAGT	>chr17:38404316+38404408
26	RPL27_8	GGACTCTGCGTCCTTGCAT	CCCAGAAACAGGCCTTGAAT	>chr17:38404389+38404468
27	FOXA1-101-49-APO	GTCCTCAGGCTTGCCACTAC	CATCTAGCAGTGGGGCATTT	>chr11:116184885+116184965
28	FOXA1-109-47	CCAAACACCGGCAAAACTAT	TGTGGCAGGGGAGCTAGTTA	>chr6:154823561+154823636
29	FOXA1-136-49	CCGGCTAGAGAGAGACACGA	AATCAGCTGGCACACACTTG	>chr17:58863608+58863705
30	FOXA3-103-32	TCCACACCCACATGTTGACT	CATTTGCTCCCAGGTGATTT	>chr6:35241643+35241721
31	FOXA3-104-28	AGCTTTTCCATTAGGACTGTGA	GCTACGGAGACCAAGTAGGC	>chr2:9714748+9714853
32	FOXA3-106-25	TGGTGCTTGGATGTTTGTGT	ACACATCTGGGACGCATTTC	>chr20:35468137+35468216
33	FOXA3-108-23	GCTCCAGACACTCTGGCTTC	GGAACAGCAAAGGCAAAGG	>chr20:44770855+44770938
34	FOXA3-110-21	TCTTGAGTCACATGGGATCTTTT	CCCGAGGGCATGTAAACTAA	>chr6:32252880+32252959
35	FOXA1-134-73	AGAGGATTGCACAACCTTTG	GCAACGCCTGTCACATTTTA	>chr2:200311871+200311958
36	FOXA1-141-26	TGACCTCTCACCGCCTTCTA	GTCAAGCAAACAAACCGTGA	>chr1:166929121+166929199
36	FOXA1-143-23	TCGCTTTTAAGTGACAGTAGTTGTG	AGAGAGAGATCAAAGGGATTCG	>chr20:5760303+5760382
38	FOXA3-100-242	GTCCAAGGGGTGCTGTTTAC	GGCTGACTGTGCAGAAACAT	>chr20:25436865+25436943
39	FOXA3-109-22	TATGTGGCATGGACTGTTGC	TAGGTTTGTGGGACCTGCTT	>chr14:33781512+33781593
